# Characterization of the tumour microenvironment phenotypes in malignant tissues and pleural effusion from advanced osteoblastic osteosarcoma patients

**DOI:** 10.1002/ctm2.1072

**Published:** 2022-10-28

**Authors:** Zhichang Zhang, Weiping Ji, Jin Huang, Yawen Zhang, Yan Zhou, Jianjun Zhang, Yang Dong, Ting Yuan, Qingcheng Yang, Xiaomin Ding, Lina Tang, Hongtao Li, Junyi Yin, Yonggang Wang, Tong Ji, Jia Fei, Bing Zhang, Peizhan Chen, Haiyan Hu

**Affiliations:** ^1^ Orthopedic Department of Shanghai Jiao Tong University Affiliated Sixth People's Hospital Shanghai China; ^2^ Clinical trial center of Shanghai Jiao Tong University Affiliated Sixth People's Hospital Shanghai China; ^3^ Pathology Department of Shanghai Jiao Tong University Affiliated Sixth People's Hospital Shanghai China; ^4^ Oncology Department of Shanghai Jiao Tong University Affiliated Sixth People's Hospital Shanghai China; ^5^ Department of Orthopaedics, Shanghai Ninth People's Hospital, School of Medicine Shanghai Jiao Tong University Shanghai China; ^6^ Department of Biochemistry and Molecular Biology Medical College of Jinan University Guangzhou China; ^7^ Orthopaedic Department of the Affiliated Hospital of Jiangxi University of Traditional Chinese Medicine Nanchang China; ^8^ Clinical Research Center, Ruijin Hospital Shanghai Jiao Tong University School of Medicine Shanghai China

**Keywords:** malignant pleural effusion, osteoblastic osteosarcoma, primary tumour, single‐cell RNA sequencing, tumour microenvironment

## Abstract

**Purpose:**

Malignant pleural effusion (MPE) is an adverse prognostic factor in patients with osteoblastic osteosarcoma; however, the cellular contexts of MPE are largely unknown.

**Experimental design:**

We performed single‐cell RNA‐sequencing (scRNA‐seq) on 27 260 cells from seven MPE samples and 91 186 cells from eight osteosarcoma tissues, including one recurrent, one lung metastasis and six primary tumour (PT) samples, to characterize their tumour microenvironment.

**Results:**

Thirteen main cell groups were identified in osteosarcoma tumour and MPE samples. Immune cells dominate the cellular contexts in MPE with more T/NK cells and less osteoclasts compared to PT samples. Of T/NK cells, CD8^+^GNLY^+^, CD8^+^KLRC2^+^ T cells and FCGR3A^+^NK cells were enriched in MPE but CD4^+^FOXP3^+^ Tregs were enriched in PT samples. Naïve IGHD^+^ B and immune regulatory IGHA1^+^ B cells were largely identified in MPE, whereas bone metabolism‐related CLEC11A^+^ B cells were significantly enriched in osteosarcoma PT. M2‐type TAMs, including CLEC11A_TAM, C1QC_TAM and Prolif_TAMs, among myeloid cells were enriched in PT, which may suppress cytotoxicity activities of T cells through multiple ligand–receptor interactions. Mature LAMP3^+^ DCs were transformed from CD1C^+^ DC and CLEC9A^+^ DC sub‐clusters when exposure to tumour alloantigens, which may improve T cell cytotoxicity activities on tumour cells under anti‐PD‐L1 treatments. In further, immune cells from MPE usually present up‐regulated glycolysis and down‐regulated oxidative phosphorylation and riboflavin metabolism activities compared to those in PT samples.

**Conclusions:**

Our study provided a novel cellular atlas of MPE and PT in patients with advanced osteosarcoma, which may provide potential therapeutic targets in the future.

## Graphical abstract

- Macrophages largely show immune‐suppressive activities and contribute to malignant pleural effusion (MPE) formation in osteosarcoma.
- Higher CD8+ T cellular proportion was noticed in MPE than in primary tumor samples; however, the CD8+ T cells show comparable cytotoxicity and exhausting activities.
- LAMP3+ dendritic cells (DCs) could be transformed from CD1c+ and CLEC9A+ DCs, which may modulate the anti‐PD‐L1 based immunotherapy activities

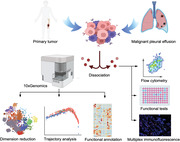

## 1 BACKGROUND

Osteosarcoma is the most common malignant bone tumour with a 5‐year survival rate of approximately 65%.^1^ Lung metastasis (LM) is the most common cause of death among patients with osteosarcoma. About 15–20% of patients have distinct metastases at primary diagnosis, and approximately 70–80% of patients have LM at advanced stage.^2^ The 5‐year survival rate of osteosarcoma patients with metastatic disease is only approximately 20–30%.^3^, ^4^ Vascular endothelial growth factor‐tyrosine kinase inhibitors (VEGF‐TKIs), such as sorafenib, regorafenib, apatinib and cabozantinib, showed clinical benefits for patients;^5^, ^6^, ^7^, ^8^ however, no significant improvement in the 5‐year survival rates was observed in patients receiving such treatments. Thus, uncovering the underlying molecular mechanisms and optimizing the management of advanced osteosarcoma patients should be performed to develop novel therapeutic approaches.

Malignant pleural effusion (MPE), with frequent symptoms of dyspnoea, cough and chest pain, is a common complication of various primary and secondary lung tumours.^9^ Under normal circumstances, a narrow lining of dynamic fluid separates the parietal and visceral pleura and maintains balance through production and reabsorption by the pleural space.^10^ In MPE, pleural membranes increased fluid production and/or reduced fluid resorption, which seriously affects the cardiopulmonary state of patients.^11^ MPE is associated with poor prognosis caused by pleural dissemination, but the corresponding biomarkers and pathophysiological mechanisms of MPE formation in patients with osteosarcoma remain unknown.^12^ A recent study has revealed complex compositions and cell–cell interactions in MPE samples using scRNA‐seq methods.^13^ In the current study, we investigated and compared the cellular heterogeneity and intercellular communications in MPE and primary tumours (PTs) in osteosarcoma patients, which may provide deeper insights into MPE formation and the tumour microenvironment (TME) characteristics of advanced osteosarcoma patients.

## 2 MATERIALS AND METHODS

### 2.1 Participants recruitment and samples collection

A total of 15 patients who were diagnosed with osteoblastic osteosarcoma were recruited at Shanghai Sixth People's Hospital affiliated with Shanghai Jiaotong University from October 2017 to November 2021. The collection of eight samples, including six PT, one recurrent tumour (RT) and one LM samples, was reported in our previous study.^14^ These patients had received standardized first‐line adjuvant and neoadjuvant chemotherapy composed of a cocktail of four drugs, including doxorubicin, cisplatin, methotrexate and ifosfamide, before the surgery treatment. For those patients with LM or RT, they received gemcitabine and docetaxel treatment before surgery. Another seven osteosarcoma patients diagnosed with MPE were recruited in the current study, and these patients had pleural metastasis confirmed by computed tomography and pathological cytological examination of exfoliated cells in the hydrothorax sample. Pleural fluid samples from individual patients were collected in heparin‐treated tubes using standard thoracentesis techniques. Pleural fluid samples were placed on ice and centrifuged at 800×*g* for 15 min, and the cells were subjected to scRNA‐seq analysis. All participants were diagnosed with osteoblastic osteosarcoma according to the NCCN Clinical Practice Guidelines in Oncology (https://www.nccn.org/), and each patient provided written informed consent. The study was approved by the ethics committee of the Shanghai Sixth People's Hospital. Two more patients who had more than 1000 ml of MPE sample agreed to donate dendritic cells (DCs) and T cells to determine the efficacy of the cytotoxicity tests in vitro. Detailed clinical characteristics of the participants are provided in Table S1.

### 2.2 Sample preparation and scRNA‐seq library preparation

Methods for the sample preparation and 3′ tag‐based scRNA‐seq of eight tumour tissue samples have been previously reported.^14^ For scRNA‐seq in seven MPE samples, the cells were resuspended gently with 1 ml phosphate‐buffered saline (PBS), and the contaminated red blood cells were further removed using RBC lysis buffer (Roche, Cat. No. #11814389001). The cells were centrifuged and washed twice with cold PBS. After staining with trypan blue (Bio‐RAD, Cat. No.#1450013), the cell cellular viability was checked under a phase contrast light microscope (Nikon, Japan). Single‐cell suspension with a concentration of 800–1200 cells/μl (90–95% viability) was loaded onto a 10× Chromium a Chip to capture a total of 8000–10 000 cells for each sample. After mixing with the barcoded gel beads on a Chromium Controller (10×Genomics), 5′‐tag‐based reverse transcription reaction was performed. After the droplets broke, the barcoded‐cDNA was purified using DynaBeads, followed by cDNA amplification for 14 cycles. After partial cDNA fragmentation and splicing, mRNA sequencing libraries of scRNA‐seq suitable for the Illumina sequencing platform were constructed. Part of the individual 5′‐tag‐based cDNA library product from MPE samples was used as templates for the construction of T cell receptor (TCR) joining (VDJ) sequencing library following the manufacturer's instructions (10×Genomics).

### 2.3 Library sequencing and data processing

The individual sequencing library was evaluated on an Agilent Bio analyser using the High Sensitivity DNA Kit (Agilent Technologies), and the libraries were pooled for DNA sequencing on the Illumina HiSeq X platform with 150‐bp paired‐end reads. Raw base call files were converted into FASTQ files using ‘mkfastq’ in the Cell Ranger software tool kit (version 2.1.1). Then, the FASTQ files were applied to generate a single‐cell gene expression matrix using the GRCh38 Ensembl build 92 genome sequences as the reference. Reads with the same cell barcode, unique molecule identifiers (UMIs) and genes were grouped together to calculate the UMIs of each gene in individual cells using the ‘count’ command. The TCR‐VDJ sequencing data produced from the Chromium Single Cell 5′‐VDJ libraries were processed using ‘vdj’ command of the Cell Ranger tool kit (version 4.1.0). After removing empty droplets, the filtered gene expression matrices were used for further analysis.

### 2.4 scRNA‐seq data integration and dimensionality reduction analysis

The filtered gene expression matrix generated by Cell Ranger (version 2.1.1) was used as the input data for the Seurat (version 4.1.1) analysis pipeline of R (version 4.1.3). The gene expression data of individual samples were processed using the CreateSeuratObject() function to create a Seurat object and the gene expression levels of the cells were normalized by the log‐normalization algorithm. Cells with nUMI < 200 or the percentage of the mitochondrial genes > 10% were dropped. Potential doublets or higher‐order multiplets were identified using the DoubletFinder package (version 2.0.2) of R with an expected doublet rate of .05 and dropped from further analysis. The top 2000 highly variable genes of the normalized expression matrix were identified, centred, scaled and used for dimension reduction. Individual Seurat objects were merged and potential batch effects were adjusted using the Harmony package (version 1.0) with default settings.^15^

### 2.5 Cellular sub‐clustering and annotation analysis

With the integrated joint embedding produced by Harmony, cellular sub‐clusters were identified using the Louvain algorithm after computing a shared nearest‐neighbour graph implemented in the ‘FindClusters’ function in Seurat. Cellular sub‐clusters were visualized in a two‐dimensional space produced using the t‐distributed stochastic neighbour embedding (t‐SNE) or uniform manifold approximation and projection (UMAP) plot. Cell sub‐clusters were annotated based on the canonical gene expression levels of well‐known markers, including tumour cells (PTH1R, CD24 and GPC1), cancer association fibroblasts (CAFs; ASPN, POSTN and ACTA2), mesenchymal stem cells (MSCs; CXCL12 and SFRP2), pericytes (PDGFRB and RGS5), osteocytes (COL1A1, CPE and MEPE), T/NK (CD3D, CD8A and NKG7), myeloid (CD74, CD14 and LYZ), osteoclasts (OCs; ACP5, MMP9 and CTSK), B and plasma B cells (CD79A, CD79B and JCHAIN), endothelial cells (ECs; vWF and PECAM1), mast cells (CPA3 and MS4A2) and red blood cells (HBB and HBA1).

For sub‐clustering analysis, we repeated the abovementioned steps, including normalization, batch effects removal, dimensionality reduction and clustering to the specific cluster derived from the overall analysis. To annotate the sub‐clusters, we identified the differentially expressed genes (DEGs) for the indicated clusters compared to the rest cells using the FindAllMarkers() function with default settings. Cellular subgroups were annotated based on well‐known markers in literature or top‐ranked DEGs.^16^

### 2.6 Differentially over‐expressed genes identification and gene ontology enrichment analysis

Significantly over‐expressed genes in indicated sub‐cluster cells compared to the other cells were identified using the Wilcoxon rank‐sum test by FindAllMarkers() function of Seurat (only.pos  =  T, logfc.threshold  =  .1 and min.pct = .1). We determined the cluster‐specific over‐represented gene ontology (GO) biological processes using the compareCluster function of clusterProfiler package (version 3.14.3) of R. We performed gene sets enrichment analysis (GSEA) with the 50 hallmark gene sets in the MSigDB database (https://www.gsea‐msigdb.org/gsea/msigdb) of the DEGs between cellular groups. The GSEA was performed with the modified competitive gene set enrichment test by Cillo et al.,which was embedded in SingleSeqGset (version 0.1.2) R package.^17^

### 2.7 Single‐cell trajectory analysis

Trajectories for CD8^+^ T cells and DCs were generated by the Monocle2 algorithm (v2.8.0) of R.^18^ Briefly, the raw UMI counts were used to create a CellDataSet object by the new CellDataSet() function with the default parameter. Genes with a mean expression < .1 were filtered out from trajectory analysis, and DEGs with *q*‐value  <  .01 between cell groups were used for dimension reduction by reduceDimension() function with parameters set as reduction method  =  ‘DDR_Tree’ and max_components  =  2. Single cells were ordered and visualized with the plot_cell_trajectory() and coloured by cell groups or pseudo time as indicated. The branch expression analysis modelling algorithm was used to identify genes with distinct gene expression between branches using the branch expression analysis modelling (BEAM) algorithm. Those DEGs with a *q*‐value  <  10^−10^ in BEAM analysis were separated into clusters and visualized with the plot_genes_branched_heatmap() function. GO biological process enrichment analyses for genes in each cluster were performed using the clusterProfiler (version 3.14.3) package of R.^15^

### 2.8 TCR repertoire analysis of T cells

The filtered contig outputs of Cell Ranger (version 4.1.0) vdj analysis pipeline, including the assembled nucleotide sequences for TCR α and β chains, the coding potential of the nucleotide sequences, CDR3 sequences, translated amino acid sequence, as well as the estimated UMI value of α or β chains for each cell, were used for the TCR analysis using scRepertoire (version 1.5.2).^19^ Only cells with sequencing data for α and β chains were retained. For any given cell barcode, only the most abundant TRA or TRB clonal types were used for further analysis. The TCR sequence of individual cells was defined by an in‐frame TCR α‐β pair, and if a unique in‐frame TCR α‐β pair was present in two or more cells, this clonal type was considered a clone. The R package STARTRAC (version 0.1.0) developed by Ren and Zhang.^20^ was used to assess the enrichment of TCR in the identified T cell sub‐clusters. The degree of clonal expansion, tissue migration and state transition of T cell clusters upon TCR tracking were determined using STARTRAC algorithms (https://github.com/Japrin/STARTRAC).^20^

### 2.9 Functional module score calculation

To determine the cellular functions of an interested subgroup, we calculated the functional score for this cell sub‐cluster using the AddModuleScore() function in Seurat (version 4.1.1). Briefly, the average expression levels of signature genes in the cluster of interest were subtracted from the aggregated expression of randomly selected control feature sets. Gene sets applied for functional module analysis, including monocytes, M1‐ and M2‐tumour association macrophage (TAM) gene signature for the myeloid cells, naïve, cytotoxicity and exhausted scores for T cells, as well as migration, differentiation, apoptosis, antigen presentation and immune regulatory scores for DCs, were derived from previous studies.^21^ The gene list applied in the functional module score calculation is provided in Table S2.

### 2.10 Multiplex immunofluorescence staining

Formalin‐fixed, paraffin‐embedded osteosarcoma tissues were sectioned into 5 μm slides, and all sections were deparaffinized, rehydrated and washed according to routine methods. After treatment with 3% hydrogen peroxide for 10 min to quench endogenous peroxidase, the slides were subjected to water‐bath heating for antigen retrieval using sodium citrate antigen repair solution (BOSTER Biological Technology, Wuhan, China). To evaluate the correlation between LAMP3^+^ DCs and CD4_FOXP3^+^ Tregs, multiplex immunofluorescence staining assays were performed using the Opal™Polaris 7‐Color Automation IHC kit (NEL871001 PerkinElmer, USA) according to the manufacturer's instructions. The primary antibodies used were anti‐LAMP3 (1:50, #12632‐1‐AP, Proteintech), anti‐CD4 (1:100, #GTX44513, GeneTex) and anti‐FOXP3 (1:100, #GTX30696, GeneTex). Multispectral images were captured and viewed using the KFBIO scan system (Jiangfeng Bioinformation, China).

### 2.11 Cellular cytotoxicity assay

Monocytes from the MPE samples were obtained by density gradient centrifugation using a lymphocyte separation medium (Histopaque®‐1077, Merck, USA). Adherent cells were cultured with AMI‐V medium (0870112DK, Gibco, USA), containing interleukin 4 (IL‐4) (50 ng/ml, Cat#AF‐200‐04‐200, Peprotech, USA) and GM‐CSF (50 ng/ml, Cat#AF‐300‐03‐200, Peprotech) for 7 days and detected the DC markers by flow cytometry (FCM). The DCs were treated with or without tumour alloantigens at a final concentration of 1 μg/ml, which was the lysate of osteosarcoma cell line 143B obtained by repetitive freeze‐thaw for five times and filtered using .45 μm pore size filters.^22^ The 143B cells were inoculated at a density of 2 million/cm^2^ and co‐cultured with T cells derived from the MPE samples in culture medium containing IL‐2 (1000 U/ml, Cat 200–02, Peprotech), anti‐CD3 antibody (50 ng/ml, 05121–25, Biogems, USA) and anti‐PD‐1 antibody (2.25 mg/ml, camrelizumab, Hengrui pharmaceuticals Co., Ltd, China).^23^, ^24^ After co‐culturing for 3 days, the cytotoxicity assay was performed in a 96‐well flat‐bottom plate at the effect/target ratio of 5:1 and 10:1. Each group was assayed in triplicate.

### 2.12 Flowcytometry assay

MPE samples were collected as described above, and the cells were first labelled using the LIVE/DEAD™ Fixable Near‐IR Dead Cell Stain Kit (#10119, ThermoFisher Scientific) to detect cell viability. The cells were multi‐stained with the indicated antibodies at room temperature for 45 min. After washing with PBS solution, the stained cells were analysed using FACS Aria III (BD Biosciences, San Jose, CA, USA) and FlowJo software (Tree Star Inc., Ashland, OR, USA). Antibodies used to determine the DC properties included anti‐CD86‐APC (BioLegend; #305220, 1:100), anti‐CD80 (CD16)‐PE/Cyanine7 (BioLegend; #305421, 1:100), PD‐L1‐PE (BioLegend; #329705, 1:100) and anti‐LAMP3(Abcam;ab111090,1:100) goat anti‐rabbit IgG‐FITC (Absin;abs20004, 1:100). Only viable cells were used for subsequent analysis.

### 2.13 Survival analysis of TARGET‐osteosarcoma database

The RNA‐seq data for the TARGET‐osteosarcoma database and the corresponding clinical data were downloaded from the National Cancer Institute Genomic Data Commons database using the TCGAbiolinks package (version 2.22.2) of R. A total of 85 patients with RNA‐seq data and following‐up information were included in the analysis. For the indicated cellular sub‐cluster, we investigated the prognostic effects of the mean expression level of cellular‐specific gene signatures in patients with osteosarcoma. We applied the receiver‐operating characteristic analysis implemented in the FindAllMarkers() algorithm of R using the parameters test.use = ‘roc’ and min.pct = .2 to identify the cellular‐specific expressed genes. Genes with an area under the curve value > .7 and percent of expression (count > 0) in the assigned cell sub‐cluster > 50% but less than 1% in other cells were recognized as the cellular‐specific gene signature. Raw data from the TARGET‐osteosarcoma RNA‐seq database were processed using the DESeq2 package (version 1.36.0) for normalization and the genes across the samples were scaled. According to the average expression level of the cellular‐specific gene signature, the osteosarcoma patients were divided into higher or lower expression groups using the optimal cut‐off point derived from the maximally selected rank statistics, which was associated with the most significant association with overall survival (OS) or event‐free survival (EFS) of the patients using the R package survminer (version 0.4.9). The Kaplan–Meier survival curve analysis was performed by survival (version 3.2‐10) and survminer packages in R, which showed the prognostic results between the high and low gene signature expression groups.^25^

### 2.14 Interactions between cell types

We determined the potential ligand–receptor (L‐R) interactions according to the expression of ligands in one cell type and their corresponding receptors in the other cell types using the CellChat algorithm (version 0.0.2; https://github.com/sqjin/CellChat).^26^, ^27^ Significant L‐R interactions were identified from the permutation tests (*p* < .05). Potential L‐R communications between cell sub‐clusters were visualized using the bubble plot. We also compared L‐R interactions and the corresponding strengths between cell populations, which were visualized using a heatmap plot.

### 2.15 Genome‐wide association studies‐related gene expression patterns

We searched the PubMed database to identify the genome‐wide association studies (GWASs) that aimed to identify the susceptibility or prognosis of osteosarcoma patients, which are listed in Table S3. We determined the gene expression patterns of the loci host or nearest genes as reported in the GWASs in cellular subgroups of scRNA‐seq data, and the z‐scaled gene expression of the indicated genes was represented as a dotplot.^28^

### 2.16 Statistical analysis

R software (version 4.1.2; www.r‐project.org) was applied to perform the statistical analysis in the current study. Continuous data were presented as mean  ±  standard deviation. The significance of the differences was determined using the unpaired Student's *t*‐test or Wilcoxon test, as indicated. The two‐sided *p* <  .05 was considered as statistically significant. To determine the enrichment of specific cell types across sample types, we calculated the observed and expected cell numbers of each cellular cluster in different sample types following the formula Ro/e = (observed/expected) as previously reported,^29^ where the expected cell number of specific cell sub‐cluster was determined using the χ^2^ test. Ro/e > 1 indicates that the cell sub‐cluster was enriched in a specific sample type.

## 3 RESULTS

### 3.1 Landscape view of TME in MPE and tumour tissues in advanced osteosarcoma patients

**FIGURE 1:**
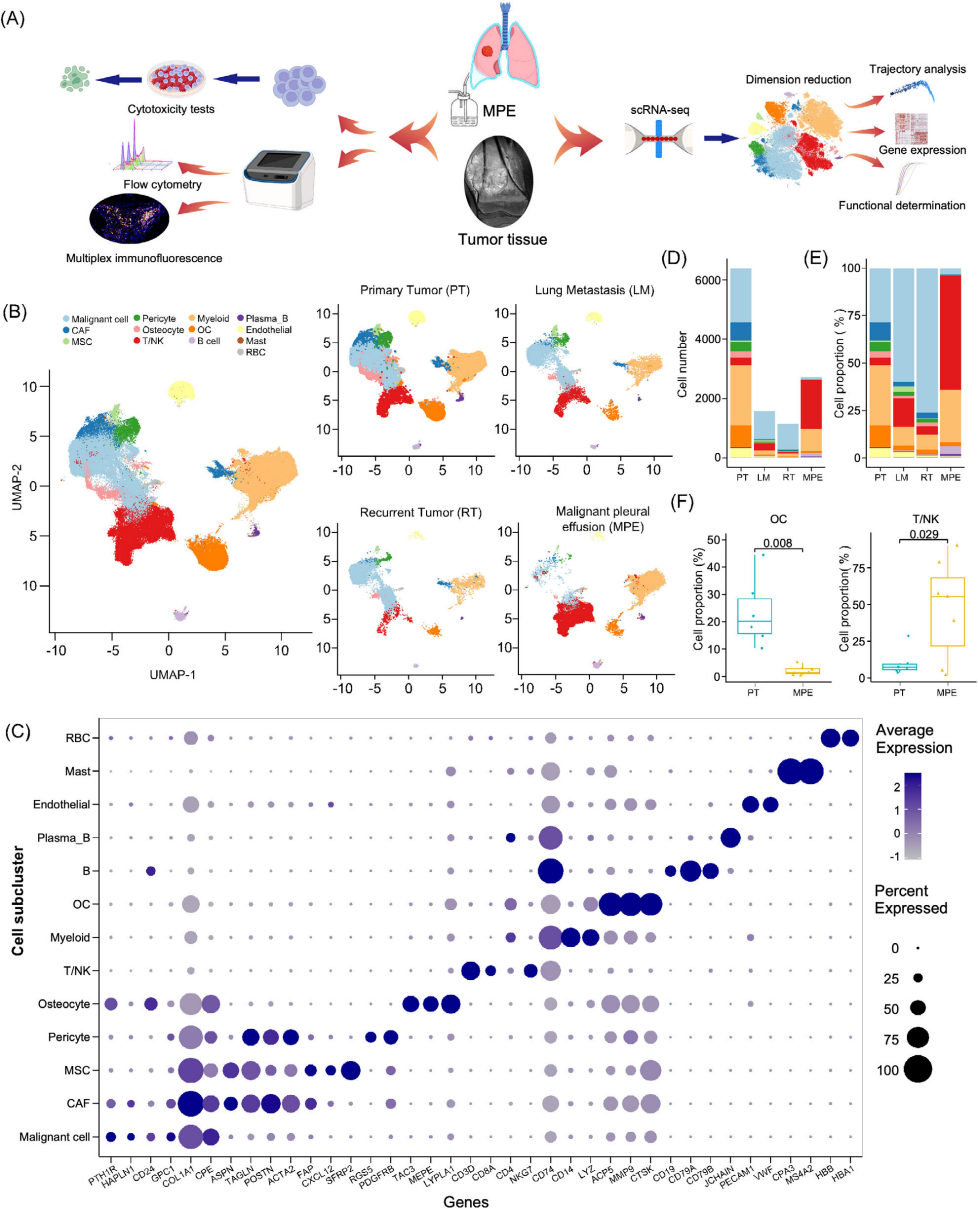
Landscape of cell clusters in tumour and MPE samples of osteosarcoma patients delineated by scRNA‐seq analysis. (A) Workflow of our study design. Eight tumour tissues and seven MPE samples from individual patients were performed for the scRNA‐seq analysis, and two other MPE samples were collected for cellular cytotoxic and flow cytometry tests in the current study. (B) The UMAP plots demonstrate the 13 mainly identified cell clusters in all OS patients or from different sample types. (C) Dotplot exhibits the expression levels of canonical markers in each cell cluster. Cell number (D) and (E) proportion of assigned cell clusters in different sample types were demonstrated. (F) The bar plot presented the relative proportion of OC and T/NK cells among immune cells (CD45^+^) between primary tumour and MPE samples. A comparison of the cellular proportion between the groups was performed using the Wilcoxon test.

Patient with MPE was associated with a poor quality of life and increased morbidity and mortality risks in patients with osteosarcoma. We collected seven MPE, six PT, one RT and one LM samples during surgical resection or perfusion drainage treatments from osteoblastic osteosarcoma patients (including six males and nine females, 11–59 years old; Table S1) to characterize their TME using scRNA‐seq methods (Figure 1A). We also determined the TCR‐VDJ sequences in five MPE samples to determine the T cell clonal status in osteosarcoma patients. After quality control, we performed UMAP and t‐SNE analysis (Figure 1B and Figure S1A) at the transcriptomic level in 118 446 cells, including 27 260 cells from seven MPE and 91 186 cells from eight tumour tissues (including PT, RT and LM samples). The cells were divided into 13 main clusters, including malignant cells (*n* = 35 418), pericytes (*n* = 3649), myeloid cells (*n* = 29 451), B cells (*n* = 1524), plasma B cells (*n* = 445), CAFs (*n* = 6680), osteocytes (*n* = 2524), OCs (*n* = 8530), ECs (ECs, *n* = 2742), MSCs (*n* = 1035), T/NK cells (*n* = 19 897), mast cells (*n* = 16) and red blood cells (RBCs, *n* = 519) based on expression levels of canonical biomarkers (Figure 1C). The total identified cellular numbers and proportions from different sample types (Figure 1D,E) and individual samples (Figure S1B,C) are provided. OCs, CAFs, MSCs, ECs and pericytes were mainly identified in tumour tissues (Figure 1D,E), whereas immune cells were enriched in MPE samples (Figure S1D). When comparing the CD45^+^ immune cell contexture between PT and MPE samples (Figure S1E), OCs were found to be enriched in PT samples, whereas T/NK lymphocytes were significantly enriched in MPE samples (Figure 1F). No significant differences in the relative proportions of B, plasma B and myeloid cells among CD45^+^ immune cells were observed between the primary tissue and MPE samples (Figure S1F).

### 3.2 Single‐cell transcriptomics atlas of T/NK lymphocytes in MPE and tumour tissues

**FIGURE 2:**
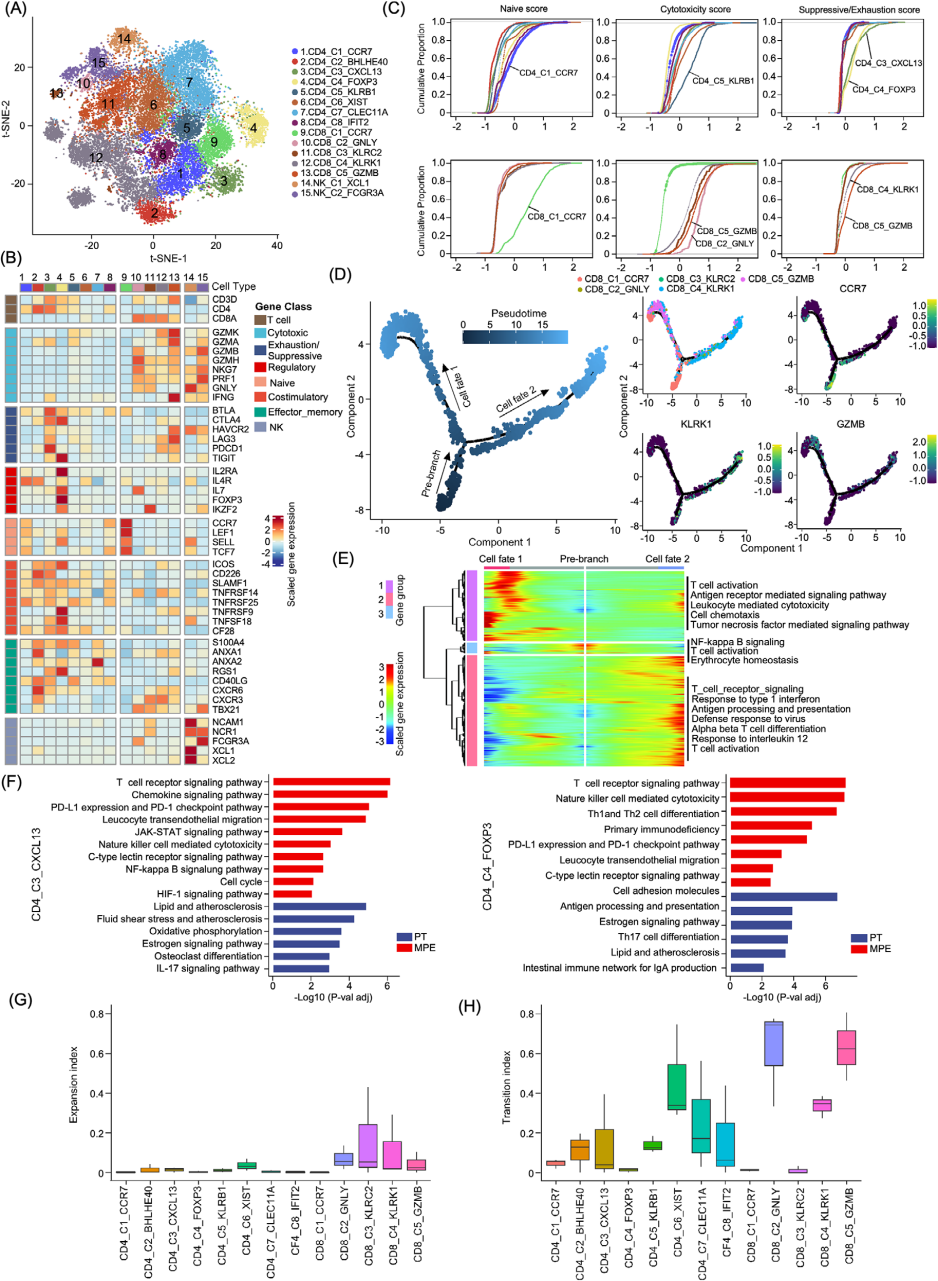
Diverse T/NK sub‐clusters in the osteosarcoma tumour microenvironment. (A) t‐SNE plot showed the CD4, CD8 and NK sub‐clusters as indicated. (B) The heatmap demonstrating the signature genes expression levels of each cellular sub‐cluster according to their functional annotation. The cellular sub‐clusters were labelled as in (A). (C) Cumulative distribution plots showed the distribution of naïve (left), cytotoxicity (middle) and suppressive/exhaustion (right) state scores in CD4^+^ T (upper panel) and CD8^+^ T (lower panel) cell sub‐clusters. A rightward shift of the curve means an increase in state scores. (D) The branch trajectory plot of the CD8^+^ T cells inferred by Monocle 2, colour from dark blue to light blue indicated the pseudotime of single cells (left panel). Each dot represents one single cell. Expression of CCR7, KLRK1 and GZMB in each cell was displayed in an inlet plot from dark blue to yellow (right panel). (E) DEGs (in rows, *q*‐value < 10^−10^) exhibited the development of CD8^+^ T cells from a naïve state into CD8_C5_GZMB (cell fate 1) and CD8_C4_KLRK1 (cell fate 2) clusters. GO terms of the DEGs in each cluster were provided. (F) The left KEGG analysis presented the functional difference in CD4_C3_CXCL13 cells between MPE and PT samples. The right KEGG analysis presented the functional difference of CD4_C4_FOXP3 Tregs between MPE and PT samples. (G) Box plots displayed the expansion index scores of all T cell sub‐clusters as indicated. The x‐axis presents cellular sub‐clusters and the y‐axis presents the expansion index. (H) The box plots displayed the transition‐index scores of all T cell sub‐clusters as indicated. The x‐axis presents cellular sub‐clusters and the y‐axis presents the transition‐index scores.

t‐SNE and UMAP clustering analyses showed that T/NK cells were subdivided into 15 subtypes, including eight CD4^+^ cell groups, five CD8^+^ cell groups and two NK cell groups (Figure 2A and Figure S2A). A total of eight CD4^+^ T cell clusters were identified (Figure 2A,B and Figure S2A). Of them, CD4_C1_CCR7 and CD4_C8_IFIT2 highly express naïve T cell markers (*CCR7*, *LEF1*, *SELL* and *TCF7*) were recognized as naïve CD4^+^ T cells (Figure 2B,C); however, CD4_C8_IFIT2 highly express the *IFIT1*, *IFIT2* and *IFIT3*, indicating they were interferon‐induced CD4^+^ T naïve cells (Figure 2B);^29^ CD4_C2_BHLHE40 sub‐cluster highly expressed *ANXA1, CD40LG*, *CXCR3* and *CXCR6*, suggesting that they were CD4^+^ T residual memory cells (CD4^+^ T_RM_; Figure 2B). CD4_C3_CXCL13 highly express *CXCL13*, *IFNG* and immune checkpoint biomarkers, including *BTLA*, *CTLA4* and *PDCD1* (Figure 2B), suggesting that these cells may be T helper 1 (T_H_1) like cells. CD4_C4_FOXP3 cells showed a high level of *FOXP3* and *IL2RA*, and they were recognized as CD4^+^ Tregs (Figure 2B). Interestingly, the CD4_C3_CXCL13 and CD4_C4_FOXP3 highly expressed immune checkpoint inhibitor receptors (e.g. *BTLA*, *CTLA4*, *PDCD1* and *TIGIT;* Figure 2B), and they showed the highest immune suppressive activities among CD4^+^ T cells (Figure 2C). CD4_C5_KLRB1 highly express the *GZMK*, *GZMB* and *CXCR6*, suggesting that these were effector memory CD4^+^ T cells (Figure 2B) and associated with high cell cytotoxicity activities among CD4^+^ T cells (Figure 2C).^30^ CD4_C6_XIST cells express *GZMK* were recognized as the effector cells in osteosarcoma TME (Figure 2B). The CD4_C7_CLEC11A highly express the central memory biomarkers, including *TIMP1*, *RGS1*, *ANXA1* and *ANXA2*, but lowly express the *CCR7* and *TCF7* (Figure 2B), indicating the transient status of these central memory cells.^29^ Cellular distribution and proportion in patients and sample types were provided (Figure S2B). Compared to PT, cellular proportions of CD4_C4_FOXP3 and CD4_C7_CLEC11A cellular proportions among all T/NK cells were reduced in MPE samples but not for the other CD4^+^ T cell sub‐clusters (Figure S2C,D). As CD4_C3_CXCL13 and CD4_C4_FOXP3 highly expressed immune checkpoint blockers, we compared the gene expression patterns between cells derived from PT tissue and MPE samples. Compared to the PT‐derived cells, both CD4_C3_CXCL13 and CD4_C4_FOXP3 cells in MPE showed enhanced activities of PD‐L1 expression and PD‐1 checkpoint pathway, TCR signalling pathway and C‐type lectin receptor signalling pathway, suggesting that their immune regulatory activities were augmented in MPE samples (Figure 2D).

Further clustering of the CD8^+^ T cells identified five sub‐clusters (Figure 2A,B), including the naive CD8_C1_CCR7 sub‐cluster characterized with high levels of naïve biomarker genes *CCR7*, *LEF1*, *SELL* and *TCF7* (Figure 2B); recently activated effector memory or effector T (Temra) cells CD8_C2_GNLY characterized with high expression of *PRF1*, *FGFBP2*, *FCGR3A*, *GZMH* and GNLY (Figure 2B); two effector memory (Tem) clusters CD8_C3_KLRC2 and CD8_C4_KLRK1 characterized with high expression levels of granzyme genes (*GZMA*, *GZMH* and *GZMK*) and *PRF1* (Figure 2B). CD8_C4_KLRK1 also highly express the immune inhibitors *CTLA4*, *PDCD1* and *LAG3*, indicating the pre‐exhausting status of these cells (Figure 2B); and the terminal exhausting CD8_C5_GZMB cell sub‐cluster was characterized with highly express the inhibitory markers (*PDCD1*, *HAVCR2*, *LAG3* and *TIGIT*; Figure 2B) as well as the cytotoxic effectors (*GZMK*, *GZMA*, *NKG7*, *PRF1* and *IFNG*).^29^, ^30^ Through evaluating the expression level of signature genes, we noticed that CD8_C2_GNLY and CD8_C5_GZMB showed the highest cellular cytotoxicity activities (Figure 2C), while CD8_C4_KLRK1 and CD8_C5_GZMB showed relatively higher levels of exhaustion activities compared to other CD8^+^ T cells (Figure 2C). It was the CD8_C2_GNLY and CD8_C3_KLRC2 rather than the other CD8^+^ T cell sub‐clusters that were significant enriched in MPE than PT samples (Figure S2D). Using the Monocle2 algorithm, we noticed a two‐branched transition trajectory of CD8^+^ T cells with CD8_C1_CCR7 as the root and two exhausted CD8_C4_KLRK1 and CD8_C5_GZMB cell sub‐clusters as terminal status (Figure 2E,F). Along with the trajectory to CD8_C5_GZMB branch (cell fate 1), T cell activation, leukocyte‐mediated cytotoxicity, cell chemotaxis and TNF‐mediated signalling pathway were enhanced, while signalling pathways related to the response to type 1 interferon, antigen processing and presentation, response to IL‐12 were enhanced along with the CD8_C4_KLRK1 branch (cell fate 2).

We performed scTCR‐seq of T cells in five MPE samples and successfully sequenced the VDJ sequences of TCR regions in 8282 out of 11 010 total T cells (Figure S3A). Clonal cells were mostly identified in CD8^+^ T cells rather than CD4^+^ T cells (Figure S3B), suggesting that the activation of CD8^+^ T cells plays vital roles in anti‐tumour activities of osteosarcoma. Using the STARTRAC algorithm, we determined the expansion and transition activities of the T cells (Figure 2G,H). CD8_C2_GNLY, CD8_C4_KLRK1 and CD8_C5_GZMB showed relatively higher expansion and transition index score (Figure 2G,H). Interestingly, the terminal CD8^+^ T sub‐clusters, including CD8_C4_KLRK1 and CD8_C5_GZMB, had the highest proportion of hyper‐expanded cells, suggesting that these cells contributed to the tumor‐responsible cytotoxicity activities by CD8^+^ T cells (Figure S3C,D). Interestingly, when we compared the cellular cytotoxicity and exhaustion activities of all CD8^+^ T cells from MPE and PT, no significant differences in the cytotoxicity or exhaustion activities were noticed, indicating the similarity anti‐tumour activities in CD8^+^ T in MPE and PT (Figure S3E). Compared the metabolic activity score of each T cell sub‐cluster between MPE and PT, we found increased activities of phenylalanine metabolism, glycolysis and gluconeogenesis, tyrosine metabolism and pentose and mannose metabolism in MPE‐derived T cells, while the riboflavin metabolism, oxidative phosphorylation and glutathione metabolism activities were decreased (Figure S3F).

Two NK cell sub‐clusters, NK_C1_XCL1 and NK_C2_FCGR3A, were identified (Figure 2A,B). Compared to NK_C1_XCL1 cells, NK_C2_FCGR3A showed stronger expression of genes related to nature killer‐mediated cytotoxicity, chemokine signalling pathway, toll‐like receptor signalling pathway, Th17 and Th1/2 cell differentiation signalling pathways (Figure S2E). Furthermore, the cellular proportion of NK_C2_FCGR3A was higher in MPE than in PT (Figure S2D), which indicated the enhancement of NK cell‐mediated cytotoxicity by NK cells in MPE samples.

### 3.3 Diversity of B cells in patients with advanced osteosarcoma patients

**FIGURE 3:**
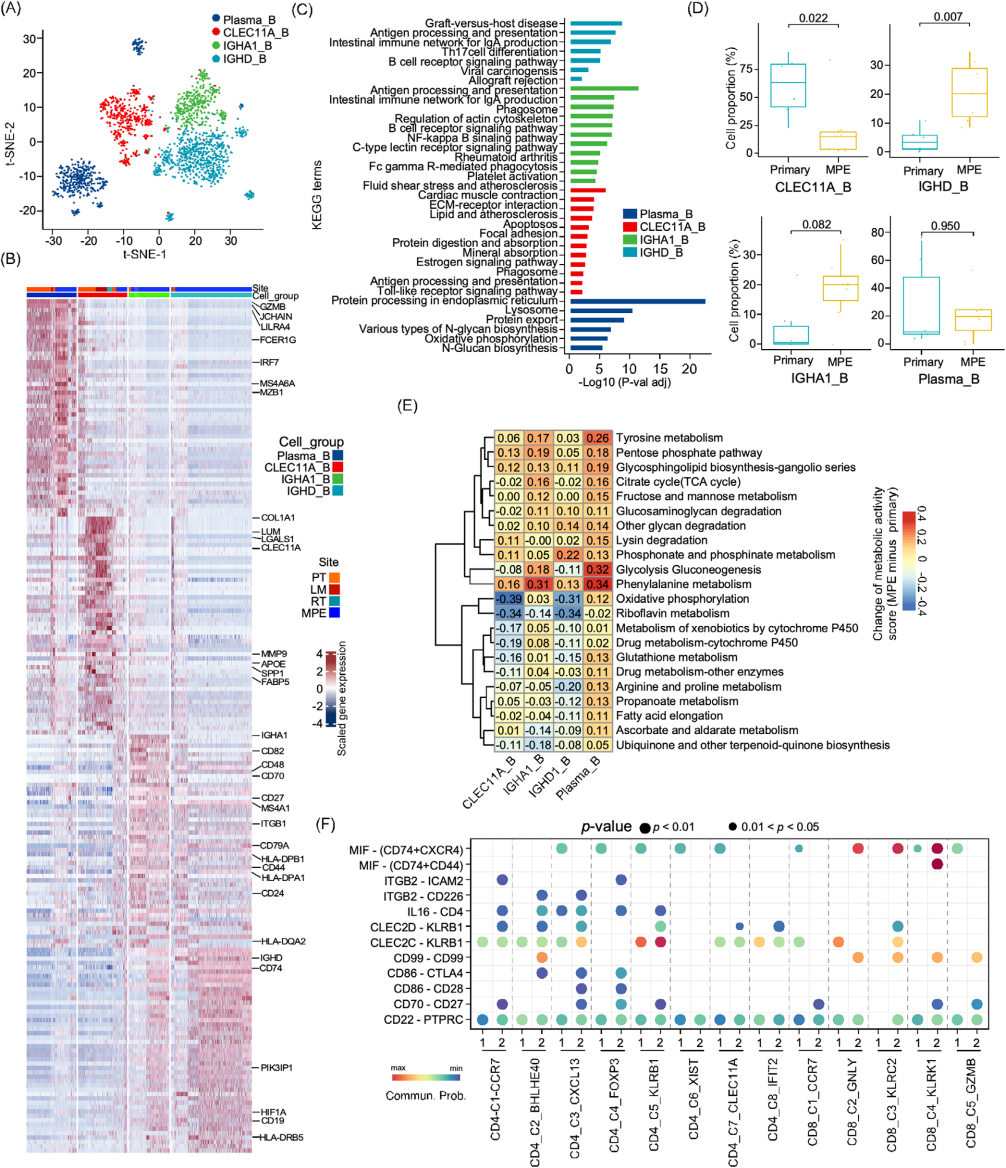
Characterization of B cells in osteosarcoma tumour tissues and MPE. (A) The t‐SNE plot clustered B cells into four sub‐clusters as coloured. (B) The heatmap showed the differentially expressed genes between four B cell sub‐clusters. (C) Bar plot indicated the KEGG enrichment analysis of over‐expressed genes in indicated B cell sub‐clusters. (D) The box plot displayed the proportion of B sub‐clusters in osteosarcoma primary tumour (PT) or MPE samples. A comparison between the groups was performed using the Wilcoxon test. (E) The heatmap presented the differences in metabolic pathway scores in B cell sub‐clusters from MPE and primary tumour tissues (MPE minus PT score). (F) Dotplot exhibited the comparison of ligand–receptor (L‐R) interactions between the IGHA1_B cells and T cell sub‐clusters in MPE and PT. 1 indicated PT and 2 indicated MPE sample.

Sub‐cluster analysis of 1,969 B cells identified four subsets, including plasma_B, CLEC11A^+^ B (CLEC11A_B), IGHA1^+^ B (IGHA1_B) and IGHD^+^ B (IGHD_B) cells, using the t‐SNE and UMAP methods (Figure 3A, Figure S4A,B). CLEC11A_B may be involved in bone metabolism as it highly expresses *SPP1*, *CLEC11A* and *MMP9* ^31^ (Figure 3B). IGHD_B was characterized by CD19^+^/IGHD^+^/CD27‐cells, which are recognized as naïve B cells (Figure 3B).^32^ IGHA1_B cells showed relatively higher expression of immune regulation genes, including *CD82, CD70, CD27, CD79A, CD44, HLA‐DPB1* and *HLA‐DPA1*, which have been suggested as regulatory B cells (Bregs; Figure 3B).^33^ KEGG enrichment analysis showed that CLEC11A_B cell over‐expressed genes were associated with extracellular matrix (ECM)–receptor interactions, protein digestion and absorption, mineral absorption and oestrogen signalling pathways, which further indicated that they may be involved in bone metabolism, osteoblastic tumour cell turnover, proliferation and invasion (Figure 3C). Compared to other B cells, IGHA1_B cells were associated with enhanced activities of antigen processing and presentation, phagosomes, B cell receptors, NF‐κB, C‐type lectin receptors and platelet activation signalling pathways (Figure 3C). CLEC11A_B was exclusively enriched in PT tissues, whereas the IGHD_B and IGHA1_B cells were enriched in the MPE samples (Figure 3D and Figure S4C). As metabolic properties may modulate immune cell activities, we compared the metabolic activities of B cells in PT and MPE (Figure 3E). We found that phenylalanine metabolism, glycolysis, pentose phosphate and tyrosine metabolism were enhanced in MPE‐derived B cell subtypes, whereas oxidative phosphorylation, riboflavin, glutathione, arginine and proline metabolism were reduced (Figure 3E). These data suggested that targeting metabolic signalling pathways may provide novel therapeutic targets for osteosarcoma treatment.

Using the CellChat algorithm, we evaluated the potential ligand–receptor (L‐R) interactions between the B and T cell sub‐clusters in MPE samples (Figure S4D). We found that IGHA1_B plays a coordinated role in T cell recruitment through the *CD22‐PTPRC/CD45* signalling axis (Figure S4D). Through the *CD70‐CD27* and *CD86‐CD28* axes, IGHA1_B may induce cellular activities of cytotoxic CD8^+^ T cells (Figure S4D). IGHA1_B cells also regulate CD4^+^ T cells through the *IL6‐CD4* and *CLEC2D/CLEC2C‐KLEB1* signalling axis (Figure S4D). IGHA_B1 cells may induce CD4^+^ T cell exhaustion, as *CD86‐CTLA4* interactions were observed between IGHA1_B, CD4_C3_CXCL13 and CD4_C4_FOXP3 cells (Figure S4D). These results may underlie the mechanisms by which immune activation is accompanied by immune suppression in T cells, as previously reported.^26^, ^34^ In the TARGET‐osteosarcoma dataset, we noticed that patients with higher IGHA1_B gene signature score were associated with better EFS and OS, suggesting tumour suppressive roles of IGHA1_B cells in osteosarcoma patients. Patients with higher plasma_B cell gene signature scores were associated with poorer EFS but with better OS, indicating the diverse roles of plasma_B cells in osteosarcoma patients (Figure S4E). We then compared the cellular interactions between IGHA1_B and T sub‐clusters between PT and MPE. Enhanced CD86‐CD28, CD86‐CTLA4 and CD70‐CD27 L‐R interactions with CD4_C3_CXCL13 or CD4_C4_FOXP3 cell sub‐clusters were observed in MPE compared to PT. Enhancement of *CD99‐CD99* and *MIF‐(CD74^+^CXCR4)* L‐R interactions between IGHA1_B and CD8_C2_GNLY, CD8_C3_KLRC2 and CD8_C4_KLRK1 cell sub‐clusters was also observed in MPE compared to PT samples (Figure 3F). These results suggested the augmented immune regulation activities by Bregs on T cells in MPE samples in contrast to PT.

### 3.4 Immunosuppressive microenvironment created by myeloid cells in osteosarcoma patients

**FIGURE 4:**
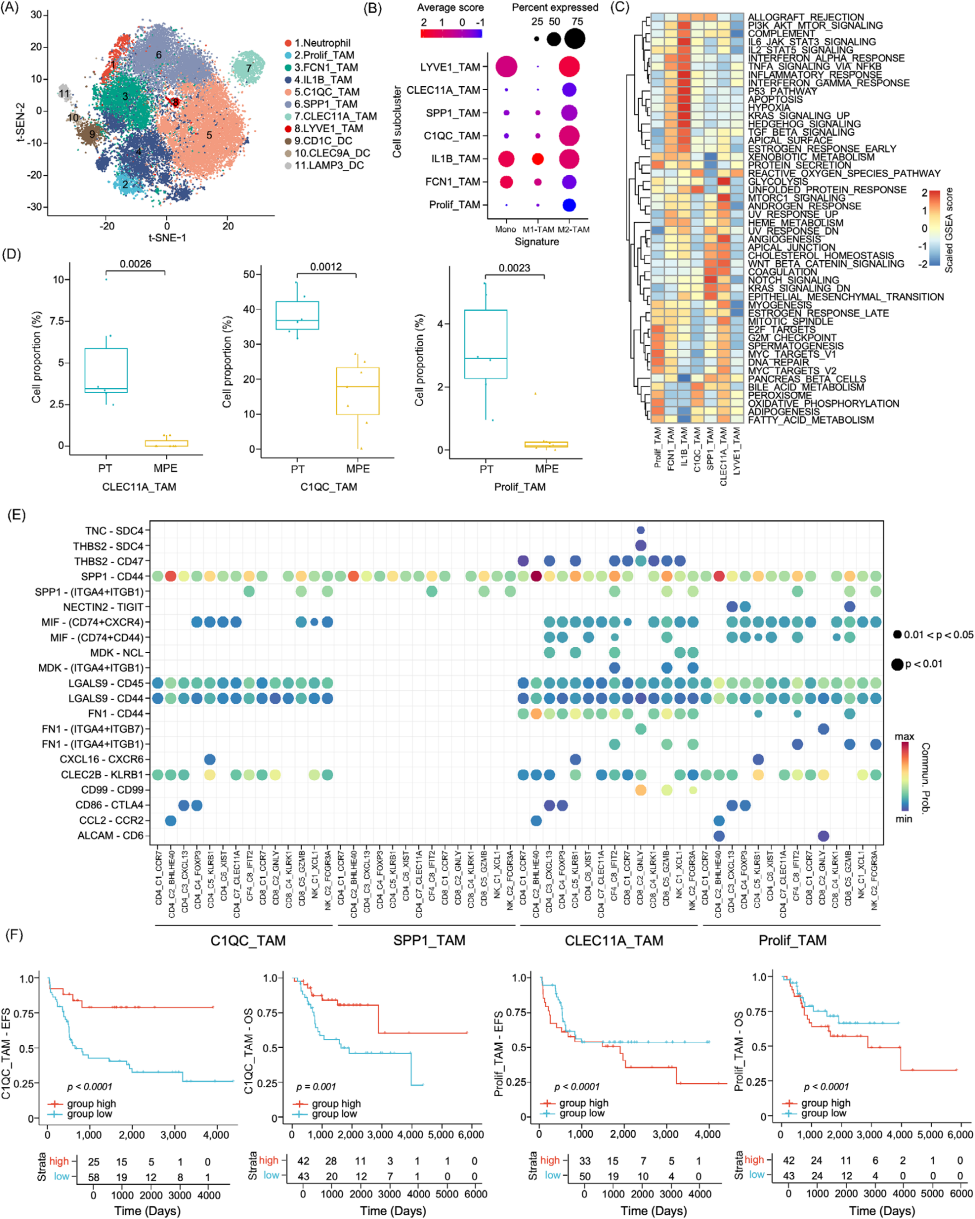
Diversity of myeloid cells in the osteosarcoma tumour microenvironment. (A) t‐SNE plot showed the 11 major sub‐clusters of myeloid cells. (B) Dot plot displayed monocyte, M1‐ and M2‐type tumour‐associated macrophage (TAM) gene signature scores in each myeloid cell type. (C) Heatmap showed the enriched hallmark gene signatures in TAM sub‐clusters. (D) Box plots presented the differences in selected TAM proportions between MPE and primary tumour (PT) samples. (E) Dotplot showed the significant ligand–receptor interactions of selected TAM sub‐clusters with T/NK sub‐clusters in PT tissues. (F) The Kaplan–Meier plot demonstrated event‐free survival (EFS) and overall survival (OS) curves of TARGET‐osteosarcoma patients according to C1QC_TAM and Prolif_TAM gene signature scores. Comparisons between groups were performed using the two‐sided log‐rank test.

Using t‐SNE clustering analysis, 29 451 myeloid cells were sub‐clustered into 11 subsets, including neutrophils, seven TAM sub‐clusters and three DC groups (Figure 4A and Figure S5A). As monocytes in the blood are induced into TAMs in TME, and the macrophages were divided into M1‐ or M2‐TAMs with anti‐tumourigenic and pro‐tumourigenic properties, we determined the gene expression signatures of monocytes and the M1‐ and M2‐type TAMs.^35^ We found that IL1B_TAM and FCN1_TAM showed relatively higher levels of monocytes and M1‐TAM signatures (Figure 4B and Figure S5B), and these cells showed higher activities of IL6_JAK_STAT3, IL2_STAT5, inflammatory response and complement‐related pro‐inflammatory signalling pathways compared to other TAMs (Figure 4C), indicating that these cells were early‐stage M1‐type like TAMs transformed from monocytes. However, IL1B_TAM and FCN1_TAM cells also positively expressed the M2‐TAM signature genes, including *MRC1*, *CD206*, *MS4A4A* and *IL4I1*, suggesting that they were undergoing the transition into M2‐TAM in osteosarcoma TME. Prolif_TAM, CLEC11A_TAM, SPP1_TAM and C1QC_TAM predominantly showed higher M2‐TAM signature (Figure 4B and Figure S5B), and were associated with higher activities in bile acid metabolism, adipogenesis, fatty acid metabolism and oxidative phosphorylation (Figure 4C).^36^ Prolif_TAM, CLEC11A_TAM, SPP1_TAM and C1QC_TAM predominantly showed higher M2‐TAM signature levels (Figure 4B and Figure S5B), and they were associated with higher activities in bile acid metabolism, adipogenesis, fatty acid metabolism and oxidative phosphorylation (Figure 4C). For M2‐TAMs, we noticed an enrichment of C1QC_TAM, CLEC11A_TAM and Prolif_TAM among the CD45^+^ immune cells in PT than in MPE samples (Figure 4D, Figure S5C,D), indicating a stronger immunosuppressive TME created by TAMs in PT tissues. LYVE1^+^ TAMs (LYVE1_TAMs) were only identified in MPE samples and have been recognized as LYVE1^high^/MHCII^low^ monocyte‐derived resident tissue macrophages alongside blood vessels which may restrain inflammation and fibrosis (Figure S5A).^37^ To further evaluate the immunoregulatory activities of TAMs, we examined the L‐R interactions between M2‐TAMs and T/NK sub‐clusters in PT (Figure 4E). We found that M2‐TAMs recruit or activate T cells through multiple signalling pathways, including *CXCL16‐CXCR6*, *MIF‐(CD74^+^CXCR4)*, *MIF‐(CD74^+^CD44)* and *SPP1‐CD44*. These cells also recruited CD4_C4_FOXP3 through *CD86‐CTLA4*, which may contribute to the immune evasion of tumour cells in osteosarcoma TME (Figure 4E). Interestingly, *NECTIN2–TIGIT* interaction was enriched in proliferating TAMs and CD4_C3_CXCL13, CD4_C3_FOXP3 and CD8_C2_GNLY cell sub‐clusters, indicating that Proli_TAM may lead to T cell exhaustion in osteosarcoma PT tissues. In the TARGET‐osteosarcoma dataset, patients with a higher proportion of C1QC_TAM were associated with a better prognosis; however, patients with higher Proli_TAM proportions were associated with poorer EFS and OS (Figure 4F).

### 3.5 LAMP3^+^ mature DCs bidirectionally regulate the T cell activities

**FIGURE 5:**
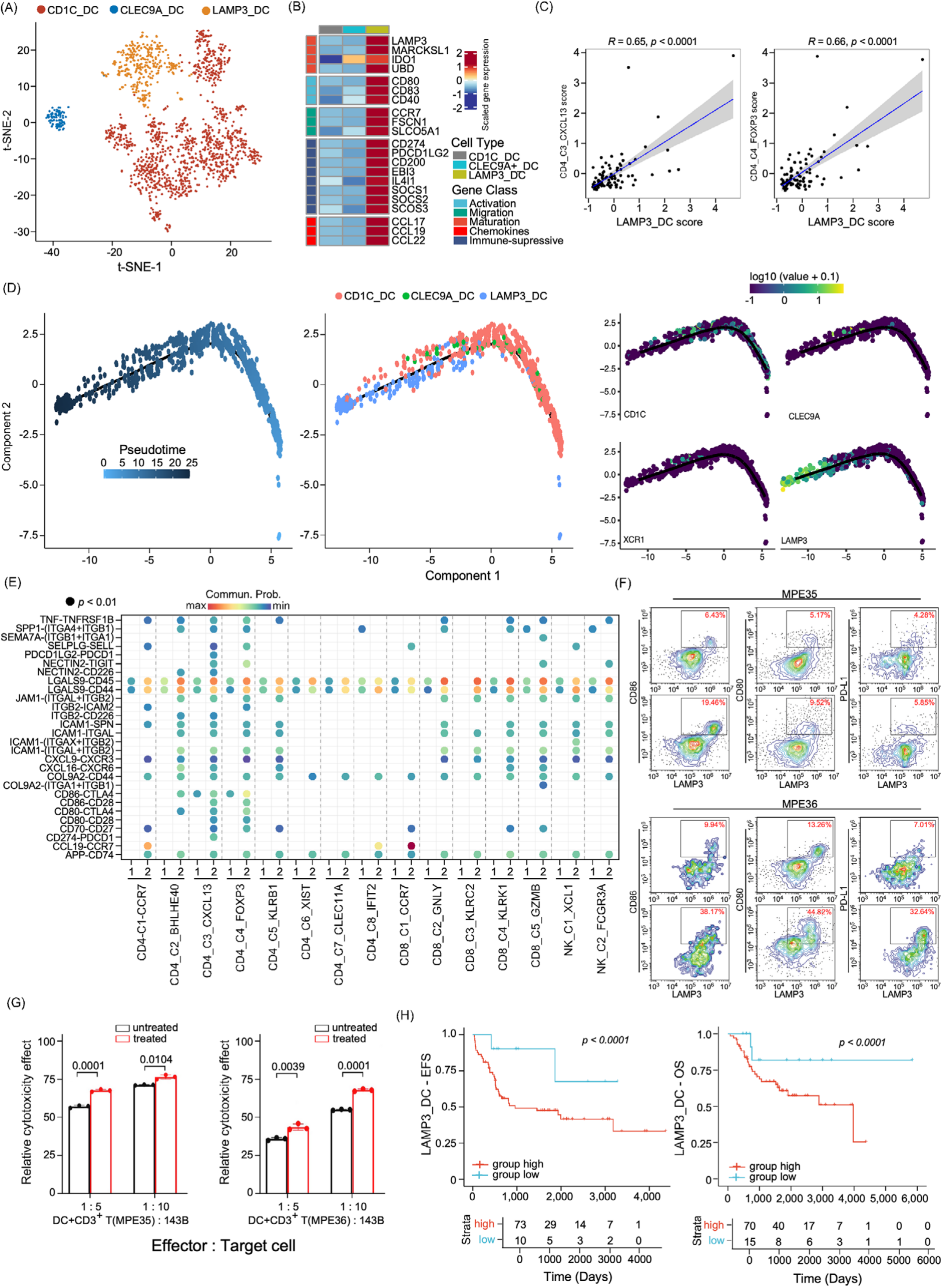
Characterization of dendritic cell (DC) sub‐clusters in osteosarcoma patients. (A) The t‐SNE plot showed the three DC sub‐clusters in osteosarcoma patients as coloured. (B) The heatmap displayed the normalized mean expression of genes associated with activation, migration, maturation, chemokines and immuno‐suppressive activities in the three DC clusters. (C) Scatter plot presented the correlation between LAMP3_DC and CD4_C4_FOXP3 Treg or CD4_C3_CXCL13 gene signature scores in TARGET‐osteosarcoma patient cohort (*n* = 85). (D) Trajectory analysis plots of DC cell functional state transition, coloured by pseudotime (left panel), cell sub‐cluster (middle panel) or the expression levels of biomarker genes, including CD1C, CLEC9A, XCR1 and LAMP3 (right panel), respectively. (E) Dotplot displayed the comparison of the ligand–receptor interaction of LAMP3_DC and with T/NK sub‐clusters in MPE and primary tumour tissues. 1 indicated primary tumour (PT) and 2 indicated MPE samples. (F) Flow cytometry plot exhibited LAMP3, CD80, CD86 and PD‐L1 levels in DCs treated with tumour alloantigens in two MPE samples (MPE35 and MPE36). The upper panel shows the untreated control group, and the lower panel shows DCs loaded with tumour alloantigens. (G) The cytotoxicity test showed a significant difference between the control and treated groups. A comparison was performed using Student's *t*‐test. (H) The Kaplan–Meier plot demonstrated event‐free survival (EFS) and overall survival (OS) of TARGET‐osteosarcoma patients (*n* = 85) categorized by the LAMP3_DC gene signature score. The x‐axis represents time (days) and the y‐axis represents survival probability. Comparisons between curves were performed using log‐rank tests.

Heterogeneous sub‐clusters were identified for DCs, which were clustered into three main subsets, including CD1C_DC, CLEC9A_DC and LAMP3_DC, according to canonical biomarkers (Figure 5A and Figure S6A). LAMP3_DC was recognized as the activated and mature DC sub‐cluster because of its relatively high expression of maturation (*LAMP3*, *MARCKSL1*, *IDO1* and *UBD*) and immune activation genes (*CD80*, *CD83* and *CD40*; Figure 5B).^38^, ^39^ Compared to the other two DC clusters, LAMP3_DC showed higher differentiation and apoptosis activities, but lower antigen processing and presentation abilities (Figure S6B). Moreover, mature LAMP3_DC also showed higher immunosuppressive activities as they highly expressed *CD274* (*PD‐L1*), *PDCD1LG2* (*PD‐L2*), *CD200*, *IDO1*, *SOCS1*, *SOCS2* and *SOCS3* (Figure 5B). LAMP3_DCs also expressed higher levels of chemokine ligands, including *CCL17*, *CCL19* and *CCL22* (Figure S6C), which are known to recruit immune cells, such as T‐regs, T helper cells and B cells through their receptors *CCR4* and *CCR7*.^40^ These results suggested that LAMP3_DC has dual immunoregulatory activity in the osteosarcoma TME. In the TARGET‐osteosarcoma cohort, we observed a strong correlation between the LAMP3_DC gene signature and the CD4_C6_FOXP3 and CD4_C5_CXCL13 cellular gene signatures (Figure 5C). Using multiplex fluorescence staining methods, we observed co‐localization of CD4^+^FOXP3^+^ Tregs and LAMP3^+^ DC in osteosarcoma tissues (Figure S6D), which indicated the recruitment of Tregs by LAMP3_DC as previous reports.^41^, ^42^ Using unsupervised trajectory analysis (Figure 5D), we identified a continuous transition of mature LMAP3_DC from CD1C_DC and CLEC9A_DC sub‐clusters, suggesting that both cDC1 and cDC2 could develop into mature LAMP3_DC in the osteosarcoma TME.

We further determined the L‐R interactions between DC_C3_LAMP3 and T cells in PT and MPE using the CellChat algorithm (Figure 5E). We found that LAMP3_DC might recruit T cells through the *CXCL9‐CXCR3* and *CXCL16‐CXCR6* signalling pathways and triggered the activation of CD4^+^ T and CD8^+^ T cells through *CD86‐CD28* and *CD70‐CD27* interactions in MPE more significantly than in PT (Figure 5E). However, higher immune checkpoint inhibitor‐mediated activities, such as *PDCD1LG2‐PDCD1*, *CD247‐PDCD1*, *NECTIN2‐CD226*, *CD86‐CTLA4* and *CD80‐CTLA4*, were also enhanced between LAMP3_DC and distinct T cell subgroups in MPE compared to PT (Figure 5E). These results suggested that LAMP3_DC simultaneously triggered T cell activation and exhaustion signalling. Compared to PT, LAMP3_DC from MPE samples showed augmented antigen processing and presentation, TCR signalling pathway, NF‐κB, TNF‐α, PD‐L1 expression and PD‐1 checkpoint signalling pathway activities (Figure S6E). As metabolic reprogramming impacts immune‐editing procession, we noticed the glycolysis and gluconeogenesis, phenylalanine metabolism, pentose phosphate pathway, fructose and mannose metabolism were increased, while riboflavin metabolism activities were decreased in DC sub‐clusters derived from MPE compared to PT samples (Figure S6F).

To clarify the immune‐regulating function of DCs, we isolated DCs from MPEs of two patients with osteosarcoma and treated them with tumour alloantigens generated from osteosarcoma cell line 143B.^43^ We noticed that the proportion of LAMP3^+^/CD86^+^ mature DC was significantly increased in the treated samples as determined by FCM analysis, confirming that LAMP3_DC could be differentiated from the other DC sub‐clusters (Figure 5F). Consistent with the scRNA‐seq results, PD‐L1 expression in LAMP3^+^ DCs also increased, along with enhanced CD80 and CD86 expression levels, compared to untreated DCs (Figure 5F). When co‐cultured with stimulated DCs and CD3^+^ T cells from the MPE samples, the cytotoxicity of T cells was enhanced when tumour alloantigen and anti‐PD‐L1 antibodies were incubated in the co‐culture system compared to unstimulated DCs (Figure 5G and Figure S6G). We found the LAMP3_DC were enriched in PT compared to MPE samples (Figure S6H). In the TARGET‐osteosarcoma dataset, patients with higher LAMP3_DC gene signatures were associated with poorer OS and EFS (Figure 5H), whereas patients with higher CD1C_DC or CLEC9A_DC proportions were associated with better OS or EFS (Figure S6I). These results indicated that tumour antigen‐stimulated LAMP3_DC may augment anti‐PD‐L1 immune therapy activities in osteosarcoma patients and may serve as a potential biomarker to identify patients who are sensitive to PD‐L1 targeting treatments.

### 3.6 Dissection of the endothelial cell sub‐clusters in osteosarcoma TME

**FIGURE 6:**
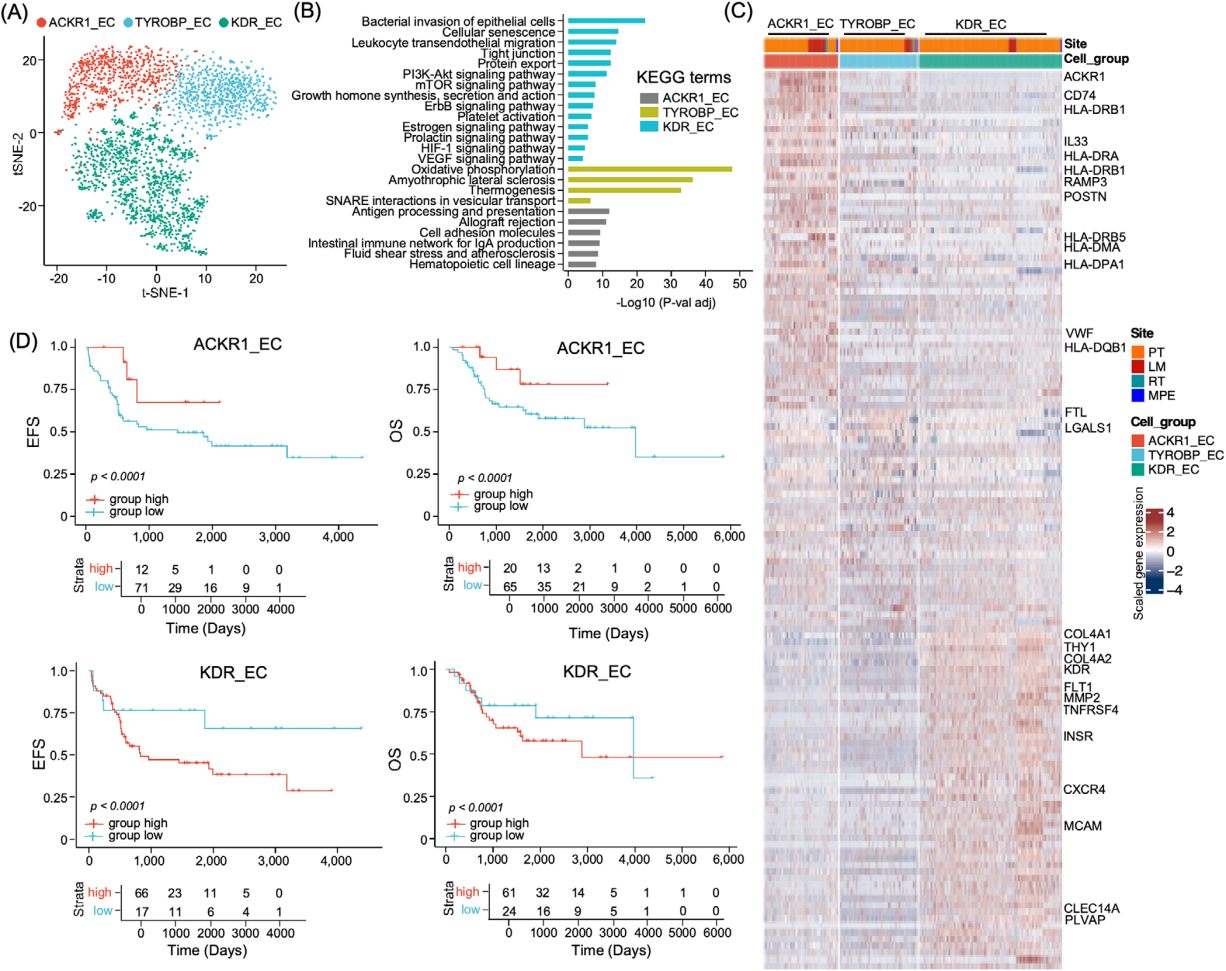
Dissection and clustering of endothelial cells (ECs) in osteosarcoma TME. (A) The t‐SNE plot showed the EC sub‐clusters in osteosarcoma TME as coloured labelled. (B) The bar plots displayed the KEGG enrichment analysis results of genes over‐expressed in indicated DC sub‐clusters compared to other sub‐clusters. (C) Heatmap presented the normalized expression of differentially expressed genes in each sub‐cluster. (D) The Kaplan–Meier curves demonstrated event‐free survival (EFS) and overall survival (OS) in the TARGET‐osteosarcoma patients cohort according to ACKR1_EC and KDR_EC gene signature. The x‐axis represents time (days) and the y‐axis represents survival probability. Comparison between the curves was performed using the log‐rank tests.

Based on t‐SNE and UMAP analyses, 2742 ECs were grouped into three subsets, including ACKR1_EC, TYROBP_EC and KDR_EC (Figure 6A and Figure S7A,B) based on classical endothelial cell markers. EC cell sub‐clusters were predominantly identified in PT samples (Figure S7C,D). KEGG analysis showed that KDR_EC was enriched in HIF‐1, VEGF and platelet activation signalling pathways (Figure 6B) as they highly expressed the VEGF receptors FLT1 (VEGFR1) and KDR (VEGFR2) and the neovascular development biomarkers PLVAP and MCAM (Figure 6C),^44^ suggesting that these cells are involved in angiogenesis of osteosarcoma TME. ACKR1_EC sub‐cluster cells showed high expression levels of *ACKR1*, which is an atypical receptor for CXCL and CCL subfamilies, including *CCL2*, *CCL5*, *CCL7*, *CXCL5*, *CXCL8* and *MCP‐1* (Figure 6C).^45^ In contrast to KDR_EC, which promotes tumour progression, ACKR1_EC may inhibit tumour cell proliferation and reduce angiogenesis through *ACKR1*‐mediated tumour suppressive signalling pathways and by sequestering cytokines. Interestingly, ACKR1_EC highly expressed MHC‐II molecules, including HLA‐DRB1, HLA_DRA, HLA‐DPA1 and HLA‐DMA (Figure 6C). KEGG and GO enrichment analysis showed that ACKR1_EC showed increased activities of antigen processing and presentation, cell adhesion molecules and the intestinal immune network for IgA production (Figure 6B and Figure S7E), indicating the immune regulatory activities of ACKR1_EC cells. In the TARGET‐osteosarcoma cohort, patients with higher ACKR1_EC gene signatures were associated with better OS and EFS than those with lower signatures, whereas patients with a higher KDR_EC signature were associated with poorer EFS and OS (Figure 6D).

### 3.7 Sub‐cluster expression of genes associated with osteosarcoma susceptibility and prognosis

**FIGURE 7:**
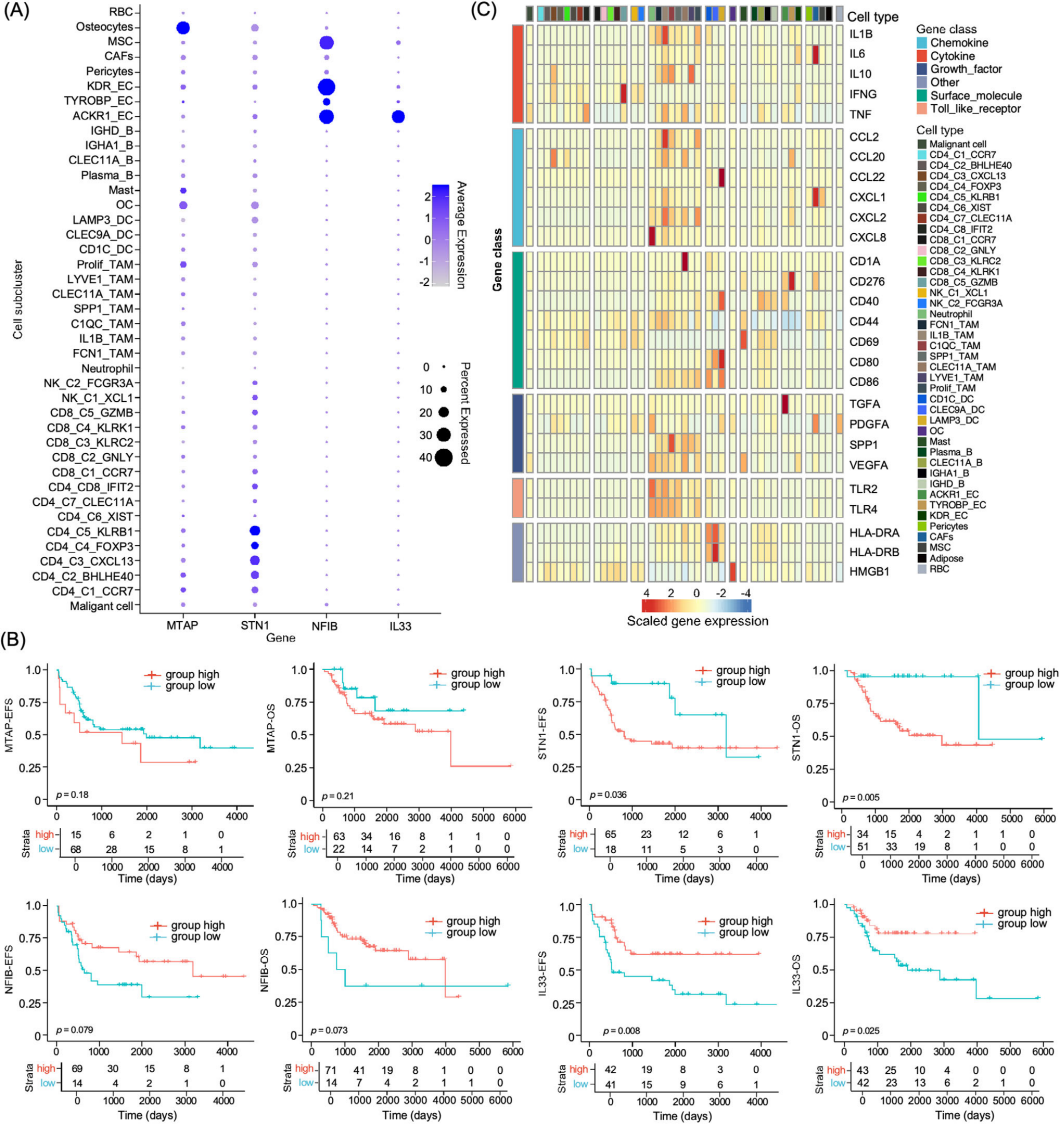
Cellular sub‐cluster‐specific expression patterns of genes related to osteosarcoma susceptibility, prognosis and MPE formation. (A) The dot plot displayed the expression of MTAP, STN1, NFIB and IL33 genes in cellular sub‐clusters from primary tumour tissues of osteosarcoma patients. (B) The Kaplan–Meier curves demonstrated event‐free survival (EFS) and overall survival (OS) in TARGET‐osteosarcoma patients with different expression levels of MTAP, STN1, NFIB and IL33 mRNA. The x‐axis represents time (days) and the y‐axis represents survival probability. (C) The heatmap presented the average expression levels of genes in each cell sub‐cluster related to MPE formation.

Several GWASs have identified novel genetic variants associated with osteosarcoma susceptibility and prognosis (Table S3).^46^ We evaluated the cellular‐specific expression of these genes in osteosarcoma tumour and the corresponding TME cells (Figure 7A and Figure S8A). We observed higher expression levels of susceptibility gene *MTAP* in osteocytes and relatively lower expression levels in CAFs and several immune sub‐clusters, including mast cells, OCs, proliferating TAMs and CD4^+^ T cells (Figure 7A); however, *MTAP* was not associated with prognosis of osteosarcoma patients (Figure 7B). *STN1* (also known as *OBFC1*), which modulates telomere length,^47^ was predominantly expressed in CD4^+^ T cell sub‐clusters (Figure 7A). Using the TARGET‐osteosarcoma cohort, we found that patients with higher *STN1* were associated with poorer OS and EFS (Figure 7B). Lower *NFIB* is significantly associated with increased osteosarcoma cell migration, proliferation and colony formation.^47^ *NFIB* was highly expressed in EC sub‐clusters and MSCs but weakly expressed in tumour cells. Lower *NFIB* levels were marginally associated with shorter OS and EFS in the TARGET‐osteosarcoma cohort (Figure 7B). Expression quantitative trait loci rs55933544 was associated with high *IL33* level as well as better OS in patients.^48^ We noticed that *IL33* was exclusively expressed in ACKR1_EC sub‐clusters (Figure 7A), and osteosarcoma patients with higher *IL33* mRNA levels were associated with better OS and EFS (Figure 7B). These results suggest that ACKR1_EC infiltration may lead to a better prognosis through IL33 secretion.^49^ GLDC is also close to rs55933544 and positively expressed in tumour cells, CD4_C4_CXCL13 cells, B cells and ECs (Figure S8A). In the TARGET‐osteosarcoma dataset, the *GLDC* mRNA level was negatively associated with the prognosis of patients with osteosarcoma (Figure S8B), indicating that rs55933544 may influence prognosis by regulating *IL33* and/or *GLDC*.^50^ *GRM4*, a susceptibility gene for osteosarcoma,^51^ is expressed in osteocytes and neutrophil but not in tumour cells (Figure S8A), and it was reported to selectively regulate *IL23* and *IL12* in myeloid cells, which promote osteosarcoma in mouse models.^52^ Osteosarcoma patients with higher GRM4 were associated with poorer EFS and OS (Figure S8B). Taken together, the cellular‐specific susceptibility and prognostic gene expression indicate that these genetic factors might influence osteosarcoma development and progression through modulating the TME.

Lymphocytes lead to vasoactive events in the pleural cavity, and MPE formation is induced by multiple chemokines, cytokines and growth factors (Figure 7C). We determined cellular‐specific gene expression levels in the MPE of patients with osteosarcoma. We found that CD8_C5_GZMB stimulates the IFN‐γ signalling pathway, whereas chemokines, cytokines and growth factors were predominantly secreted by macrophages in MPE (Figure 7C), indicating that macrophage‐targeting therapeutic methods may reduce MPE formation in the future.

## 4 DISCUSSION

Pulmonary metastasis of osteosarcoma results in pleural thickening and calcification, often leading to MPE. Combined with our previous studies,^53^ we comprehensively characterized the TME in both PT and MPE at the single‐cell level. Our results showed that the cell types in MPE were similar to those in tumour tissues; however, the cellular proportions, characteristics and functions were largely different. Overall, a high proportion of tumour cells, stromal cells and endothelial cells were enriched in PT, while the immune cells were more abundant in MPE samples. The proportion of T/NK cells, especially CD8_C2_GNLY, CD8_C3_KLRC2 and FCGR3A^+^NK cells, with strong cytotoxic activities were significantly higher in MPE than in PT samples. CD4^+^ Tregs and CD4^+^/CXCL13^+^ T cells were predominantly identified in tumour tissues, demonstrating that malignant cells are the key elements that induced immune‐tolerance. Notably, we noticed a CD4^+^ T cell sub‐cluster CD4_ C5_KLRB1 cells presented strong cytotoxicity in osteosarcoma TME. Multiple CD8^+^ T cells were identified in the osteosarcoma MPE and PT. Trajectory analysis of CD8 cells showed that naïve CD8_C1_CCR7 could differentiate into two terminal cells with higher expression levels of GZMB and KLRK1. Both sub‐clusters had high proliferation activity accompanied by exhaustion, indicating that CD8^+^ T cells became exhausted when activated.^54^ Tumour‐infiltrating lymphocytes (TILs) have been recognized as biomarkers and fascinating therapeutic approaches for numerous types of tumours, including osteosarcoma. CD8^+^ T cells play central roles in anti‐tumour immune surveillance owing to their specific cytotoxicity activities.^55^, ^56^ Zhou et al. performed a combination therapeutic method, including TILs and anti‐PD1 therapies, which improved the prognosis of patients with chemotherapy‐resistant metastatic osteosarcoma.^57^ When compared the exhausting and activation activities of all CD8^+^ T cell in MPE and PT, no significant differences were observed. As MPE samples are easier to be obtained than TILs in advanced osteosarcoma patients, MPE samples may be a potential source for adoptive CD8^+^ T cell therapy in osteosarcoma patients; however, clinical studies are warranted to evaluate the anti‐cancer activities of CD8^+^ T cells derived from MPE samples.

B cells are critical adaptive immune response cells in TME in cancers; however, the roles of B cells in osteosarcoma are largely unknown. A pioneer scRNA‐seq study performed by Liu et al. in the treatment naïve osteosarcoma patients identified three B high‐quality sub‐clusters, including the naïve, memory and plasma B cells.^58^ Zhang et al. reported that the cellular proportion of naïve B cells was significantly lower in high immune score tissues as determined using deconvolution algorithm.^59^ In the current scRNA‐seq study of advanced osteosarcoma tissues, we noticed four main B cell sub‐clusters, including the conventional naïve B cells, regulatory B cells, plasma B cells and a novel CLEC11A^+^ B cell sub‐cluster. CLEC11A (C‐type lectin domain family 11 member A), also known as osteolectin or stem cell growth factor‐β, can promote bone formation through stimulating the differentiation of mesenchymal progenitor cells into osteoblasts^60^ and maintain the adult skeleton age‐related bone loss and fracture repair.^61^ CLEC11A could recruit the endothelial cells in lung cancer^62^ and neutralization of CLEC11A reduced metastasis and viability of cancer stem cells in hepatocellular carcinoma.^63^ A recent study performed by Sun et al.^64^ in the TNF‐transgenic (TNF‐Tg) rheumatoid arthritis mouse model has purified the CD19^+^ B cells from bone marrow (TNF‐Tg BM) or subchondral bone marrow (TNF‐Tg SBM) and compared their transcriptome profiles between the cell groups. In B cells from TNF‐Tg SBM, genes related to extracellular matrix remodelling, such as ECM1, COL3A1, CTSK and LUM, were significantly increased, which were also noticed in CLEC11A^+^ B cells in our current scRNA‐seq data. We also noticed a relatively higher expression of inflammatory genes, including CXCL3, CCL4, CCL3 and SPP1 (data not shown), as for B cells in TNF‐Tg SBM in CLEC11A^+^ B cellular sub‐clusters compared to IGHA1^+^ or IGHD^+^ B cells.^64^ Interestingly, B cells in TNF‐Tg SBM also show increased levels of C‐type lectin domain family gene CLEC4D and CLEC4E, suggesting that the CLEC11A^+^ B cells in osteosarcoma may have similar biological functions as the TNF‐Tg SBM‐derived B cells.^64^ Sun et al. found that CCL3 and TNF secreted by TNF‐Tg SBM B cells may inhibit osteoblast differentiation, and B cell depletion therapy increases the numbers of osteoblasts and reduces the osteoclasts in TNF‐Tg mice.^64^ We also noticed significant L‐R interactions of TNF‐TNFRSF1B between CLEC11A_B and the T cell sub‐clusters (Figure S4D). These results indicate that CLEC11A^+^ B cells may influence the osteosarcoma progression through modulating the tumour cells, osteoclasts or T cell sub‐cluster activities in osteosarcoma, and CLEC11A_B cells may act as novel therapeutic targets in the future.

Macrophages are major TME ingredients in multiple cancers, which showed diverse biological functions. In primary osteosarcoma tissues, we noticed six macrophage subsets, while an additional LYVE1^+^ interstitial macrophage subset was noticed in MPE samples.^65^, ^66^ Among TAM subsets, IL1B_TAM and FCN1_TAM resemble M1‐like macrophages, and these TAMs showed enhanced inflammatory signalling pathways, including IL6/JAK/STAT3, IL2/STAT5, TNFα and interferon‐γ, as suggested by GSEA analysis. These inflammatory‐related TAMs may recruit and regulate immune cells during MPE formation or tumour‐associated inflammatory response through secreting IL1B, CCL2, CCL20 and CXCL2.^67^ Other TAM sub‐clusters, including CLEC11A_TAM, SPP1_TAM and C1QC_TAM, displayed M2‐type TAM properties with distinct enrichment of gene signalling pathways. M2‐type TAMs play crucial roles in the suppression of T cell‐mediated anti‐tumour immunity.^68^ By L‐R interaction analysis, these M2‐type macrophages may regulate T cell activities through multiple signalling pathways, including CD86‐CTLA4, SPP1‐CD44, CLEC2B‐KLRB1 and LGALS9‐CD44, which may provide novel immune therapy targets for osteosarcoma. Interestingly, we noticed a Prolif_TAM sub‐cluster, which is characterized with high proliferating activities and M2‐type TAM characteristics, and itwas associated with poorer prognosis of osteosarcoma patients. Proli_TAMs showed immunosuppressive activities on T cells through *NECTIN2‐TIGIT* and *CD86‐CTLA4*, and it may also regulate CD8_C2_GNLY proliferating through ALCAM‐CD6 axis.^69^ Proli_TAMs were noticed in multiple cancer types, including colorectal cancer, gastric cancer, prostate cancer and ovarian cancer, which may be generated from different ontogenies in different tissues. Whether these proliferating cells act at a transient state that may transform into other functional TAM subsets or as precursors and/or functioning as precursors need to be addressed with more studies.

Osteosarcoma has been classified as a cold tumour with a relatively poor response to immune checkpoint‐based therapy.^70^, ^71^, ^72^, ^73^ Several studies have proposed that enhancing antigen processing or presentation may improve immunogenic T cell antigens to eradicate cancer.^74^, ^75^, ^76^, ^77^ With the DC‐based vaccine loaded with Hsp70‐PCs, allogeneic CTLs exhibited strong cytotoxic activities against osteosarcoma cell lines.^78^ In the current study, three DC subsets were identified, including CD1C^+^, CLEC9A^+^ and LAMP3^+^ DCs, which is consistent with previous findings.^79^, ^80^, ^81^ Using unsupervised trajectory analysis, we noticed mature LAMP3^+^ DCs could be transformed from CD1C^+^ and CLEC9A^+^ DCs, with high expression of the classical markers CD80 and CD83, the migration marker CCR7, and the lymphocyte recirculation chemokines CCL19 and CCL22. LAMP3_DCs in MPE samples presented high immunoregulatory ability, especially with FOXP3^+^ Tregs and CXCL13^+^ T cells. Mature LAMP3_DC highly expressed CD80 and CD86, which may modulate CXCL13^+^/CD4^+^ and FOXP3^+^/CD4^+^ Tregs activities by interacting with *CD80‐CD28*, *CD80‐CTLA4*, *CD86‐CD28* and *CD86‐CTLA4*. Tumour cells induce DCs to secrete TGF‐β and stimulate FOXP3^+^/CD4^+^/CD25^+^ Tregs proliferation.^82^, ^83^ Correlation between LAMP3_DCs and FOXP3^+^ Tregs was observed in primary osteosarcoma tumour tissues, suggesting that LAMP3_DC may contribute to immune tolerance in osteosarcoma tumours through recruiting and regulating FOXP3^+^ Tregs. Immunotherapies constitute critical therapeutic approaches to overcome immune evasion and enhance therapeutic efficacy.^84^ When DCs derived from MPE samples were treated with 143B tumour alloantigen, an enhancement of cytotoxicity activities of T cells co‐cultured with anti‐PD‐L1 antibodies was noticed, which indicates that targeting DCs may facilitate the T cells‐based immunotherapy treatments.^85^, ^86^, ^87^

Our study has several limitations that should be acknowledged. First, the tumour tissues and two MPE samples were subjected to the 3′‐tagged scRNA‐seq method, whereas the other five MPE samples were subjected to 5′‐tagged scRNA‐seq along with scTCR‐VDJ sequencing analysis. This may induce potential batch effects in scRNA‐seq data analysis. We applied the Harmony algorithm for batch effects adjustment, and a well concordance of cell distribution in the dimension reduction plots was observed, indicating minimal batch effects in the study. Second, the regulatory mechanisms underlying the cellular transition between cell sub‐clusters, such as the CD8^+^ T cells and DCs, needed to be addressed. Multi‐omics methods, such as the scATAC‐seq that identifies the transposase accessible chromatin profiles in single cells, may further uncover the underlying mechanisms of cellular differentiation, polarization and activation of these cells, which may provide novel therapeutic targets. Third, only in vitro studies of DC‐based immunotherapy have been performed, in vivo studies are warranted to demonstrate the therapeutic values of DCs for osteosarcoma. At least but not last, the origins of immune cells, such as T or macrophage cells in MPE, still need to be addressed. T cells in MPE may be derived from multiple origins, including the leaked blood cells and the lung metastasized tumour samples. A recent study performed by Huang et al. evaluated the composition and functional states of infiltrating immune cells in MPE samples of non‐small cell lung cancer patients.^13^ They found a small fraction of TCR clonotypes shared by MPE and matched blood samples, indicating that T cells in MPE could partially be derived from the blood samples;^13^ however, this study did not compare the TCR clonal types of T cells in PTs or the lung metastasized tumours. Thus, the sources of the T cells in MPE need to be addressed with more TCR clonal analysis at the single‐cell level in MPE samples together with paired blood samples, lung or pleural metastasized samples and PT samples.

## 5 CONCLUSIONS

In summary, the current study described the multi‐cellular ecosystem of osteosarcoma tissues and MPE samples to gain deeper biological insights into osteosarcoma development and progression. Our data provided valuable resources for potential MPE‐based innate and adaptive immune therapeutic strategies in the future.

## CONFLICT OF INTEREST

The authors declare that there is no conflict of interest.

## Supporting information



Supplementary material

Supplementary material

Supplementary material

Supplementary material

Supplementary material
