# Supplementary material for: Characterization of the tumour microenvironment phenotypes in malignant tissues and pleural effusion from advanced osteoblastic osteosarcoma patients
Source: Clin Transl Med. 2022 Oct 28;12(11):e1072. doi: 10.1002/ctm2.1072 (PMC9615475; doi:10.1002/ctm2.1072)
Supplement: Supplementary file 1 — Supplementary material [file CTM2-12-e1072-s005.docx]

**Supplementary Information**

**Fig S1. Single-cell transcriptional profiling of cell clusters in tumor tissue and MPE samples of osteosarcoma. A,** t-SNE plot showed 13 mainly identified cell subclusters in primary tumor (PT) tissues and malignant pleural effusion (MPE) samples. Bar plot displayed the proportion (**B**) and total identified cell number (C) of each cellular subclusters from individual patients. The cellular subcluster was color labelled as indicated. D, Sample specific enrichment analysis of the 13 mainly identified cell subsets estimated by Ro/e score. E, Sample enrichment of CD45+ cell subsets estimated by Ro/e score. F, Box plot presented the cellular proportion of B cells, myeloid cells, plasma cells, mast cells and red blood cells (RBC) among the CD45+ immune cells from PT tissues and MPE samples. Comparison between the groups was performed using Wilcoxon test.

**Figure S2. The Single-cell transcriptional profiling of T/NK cells from tumor tissues and MPE samples.** A, The total and sample types stratified UMAP plots identified 15 T/NK cell sub-clusters in OS patients as indicated. B, The bar chart displayed total sub-clusters cellular numbers and their corresponding proportions in each sample or types of samples. C, Sample type enrichment analysis of T and NK cell subsets estimated by Ro/e score. D, Box plot showed T/NK cellular subcluster proportions in MPE and tumor tissues. Comparison between the groups was performed using Wilcoxon test. E, The bar chart demonstrated KEGG enrichment terms of the genes overexpressed in NK_C2_FCGR3A versus NK_C1_XCL1.

**Figure S3. Characteristics of T/NK cell subclusters in osteosarcoma samples.** t-SNE plots of T cells identified by scRNA-seq analysis, colored by TCR expansions cloning status (A) and groups (B). Cumulative bar chart of the cell number (C) or proportion (D) of specific TCR clonal status groups in each T cell sub-clusters. E, Cumulative distribution plots displayed cytotoxicity (upper panel), and exhausting (lower panel) score of CD8+ T cells in MPE and PT tissues. A rightward shift of the curve means increasing of state scores. Red line, MPE samples; Black line, primary tumor samples. Comparison between the groups was performed using Student’s t-test. F, Heatmap showed the change of metabolic activities of T/NK cell subclusters between MPE samples and PT tissues. Value in box indicate the change of metabolic score (MPE minus primary tumor). Positive (red) value indicates the enhanced activities while the negative (blue) value indicates decreased activities of all T/NK cell analysis between tumor tissue and MPE.


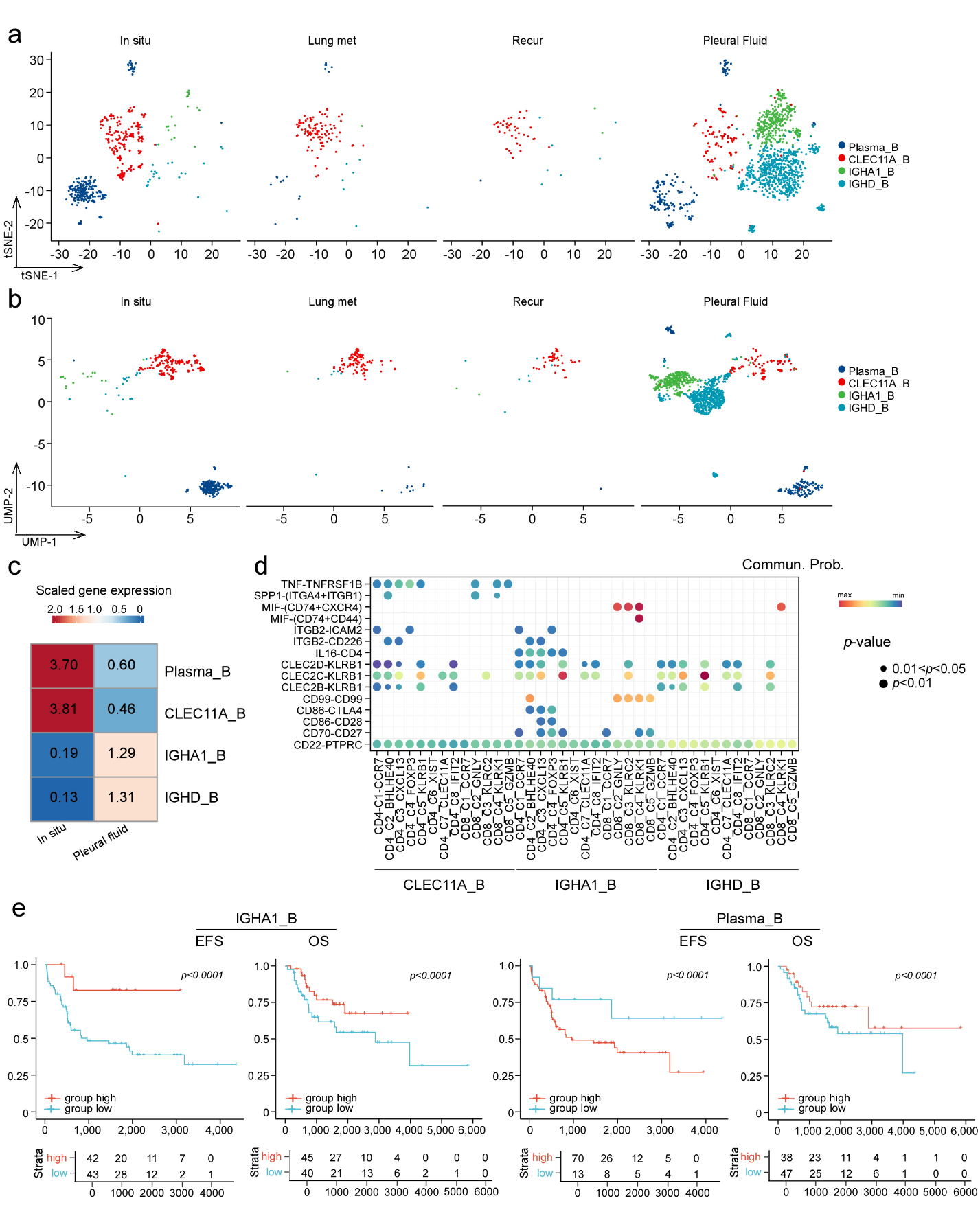


**Figure S4. Transcriptomic profiling of B cells in osteosarcoma.** The stratified t-SNE (a) and UMAP (b) plot showing the B subclusters in different sample types. Each dot represents a single cell. c, Sample type prevalence of B cell subsets estimated by Ro/e score in primary tumor (PT) or MPE samples. d, The dotplot displayed the L-R interactions between B and T cell subclusters in MPE samples determined by CellChat algorithm. e, The Kaplan-Meier plots for the event-free survival (EFS) and overall survival (OS) of patients in TARGET-osteosarcoma cohort (n = 85) categorized by the IGHA1_B and Plasma_B gene signature score. The x-axis represents time (days) and the y-axis represents survival probability. Comparisons between the survival curves were performed using the log-rank tests.

**Fig S5. Dissection of myeloid cells in osteosarcoma TME.** A, The stratified t-SNE plot shows myeloid cell subclusters as colored in different sample types. Each dot represents a single cell. PT, primary tumor; LM, lung metastasis; RT, recurrent tumor; MPE, malignant pleural effusion. B, Ridge plot showing the distribution of monocytes, M1-type tumor associated macrophage (TAM) and M2-type TAM related gene signature scores in the TAM subclusters. C, Sample type prevalence of B cell subsets estimated by Ro/e score in primary tumor (PT) or MPE samples. D, Box plot showing the proportion of selected myeloid cells in PT tissues and MPE samples. Comparison between groups was performed using Wilcoxon test.

**Figure S6. Characteristics of DC cell subclusters.** A, The UMAP plot shows the DC subclusters in color. Each dot represents a single cell. B, Box plot showing differentiation, apoptosis, antigen presentation, and immune-regulatory scores of the three DC subclusters. Comparison between groups was performed using Wilcoxon test. C, The violin plots display the normalized expression of LAMP3, CCL22, CCL19 and CCL17 in all osteosarcoma TME cell subclusters. LAMP3+DCs highly expressed CCL22, CCL19 and CCL17. D, Multiplex immunofluorescence staining of CD4, FOXP3 and LAMP3 in primary tumor (PT) tissues. CD4 is labeled in orange, FOXP3 in red, LAMP3 in green, the nucleus in blue. The scale bar represents 35 μM. E, Bar plot showing the KEGG enrichment analysis of the genes overexpressed in LAMP3+ DC from MPE samples or primary tumor (PT) tissues. F, Heatmap chart showing the change in metabolic activities in three DC subclusters between MPE and primary tumor (PT) samples (MPE minus PT). G, Morphological image showing the T cell cytotoxicity activities on 143B cells under an inverted microscope. H, Enrichment analysis of DC cell subsets estimated by Ro/e score in PT or MPE samples. **I,** Kaplan-Meier plots for event-free survival (EFS) and overall survival (OS) of TARGET-osteosarcoma patients (n = 85) categorized by CD1C_DC and CLEC9A_DC gene signature scores. The x-axis represents time (days) and the y-axis represents survival probability. Comparisons between curves were performed using log-rank tests.

**Figure S7. Characteristics of endothelial cells in osteosarcoma tumor microenvironment. A,** Stratified t-SNE plot showing the endothelial cell (EC) subclusters in different sample types. Each dot represents a single cell. **B,** Stratified UMAP plot showing EC sub-clusters in different sample types. Each dot represents a single cell. **C,** Cumulative bar chart showing the total cellular number and the relative cellular proportion (D) of endothelial cells identified in each sample type. **E,** Bar plot shows the GO enrichment analysis of the genes overexpressed in each EC sub-cluster compared to other cell groups. PT, primary tumor; LM, lung metastasis; RT, recurrent tumor; MPE, malignant pleural effusion.

**Figure S8.** A, The dot plot displayed the expression of GRM4 and GLDC genes in cellular subclusters from primary tumor tissues. B, The Kaplan-Meier plot for event-free survival (EFS) and overall survival (OS) of TARGET-osteosarcoma patients (n = 85) categorized by the GRM4 and GLDC gene expression level. The x-axis represents time (days) and the y-axis represents survival probability. Comparisons between curves were performed using log-rank test.
