# Supplementary material for: Characterization of the tumour microenvironment phenotypes in malignant tissues and pleural effusion from advanced osteoblastic osteosarcoma patients
Source: Clin Transl Med. 2022 Oct 28;12(11):e1072. doi: 10.1002/ctm2.1072 (PMC9615475; doi:10.1002/ctm2.1072)
Supplement: Supplementary file 2 — Supplementary material [file CTM2-12-e1072-s003.docx]

**Supporting Information**

*S1 Funding Information*

This work was supported by the National Natural Science Foundation of China (No. 8207101854, 82072967and 82102866), National Key Research Project of Science & Technology Ministry of China (No. 2021YFC2400600), Natural Science Foundation of Shanghai (No. 20ZR1434100), Research Project of Science & Technology of Shanghai (No. SHDC22017101 and SHDC12017X02) and Shanghai Pujiang Program (No.21PJD051), the Shanghai Shenkang Three-Year Action grant (No. SHDC2022CRS031), Science Foundation of Shanghai Sixth People’s Hospital (No. ynqn202114).

*S1 Author list*

Zhichang Zhang*^, ^[[1]](#footnote-1)^,2^, Weiping Ji*^, 1^, Jin Huang*^, 3^, Yawen Zhang^4^, Yan Zhou^4^, Jianjun Zhang^4^, Yang Dong^1^, Ting Yuan^1^, Qingcheng Yang^1^, Xiaomin Ding^4^, Lina Tang^4^, Hongtao Li^4^, Junyi Yin^4^, Yonggang Wang^4^, TongJi^5^, Jia Fei^#,6,^, Bin Zhang ^#, 7^, Peizhan Chen^#, 8^, and Haiyan Hu^#, 2,4^

1. *Orthopedic Department of Shanghai Jiao Tong University Affiliated Sixth People's Hospital, Shanghai 200233, China.*
2. *Clinical trial center of Shanghai Jiao Tong University Affiliated Sixth People’s Hospital, Shanghai, 200233, China*
3. *Pathology Department of Shanghai Jiao Tong University Affiliated Sixth People's Hospital, Shanghai 200233, China*
4. *Oncology Department of Shanghai Jiao Tong University Affiliated Sixth People's Hospital, Shanghai 200233, China.*
5. *Department of Biochemistry and Molecular Biology, Medical College of Jinan University, 601 Western Huangpu Avenue, Guangzhou 510632, China.*
6. *Department of Orthopaedics, Shanghai Ninth People's Hospital, School of Medicine, Shanghai Jiao Tong University, Shanghai, 200011, China.*
7. *Orthopaedic Department of the Affiliated Hospital of Jiangxi University of Traditional Chinese Medicine, Nanchang, 330006, China*
8. *Clinical Research Center, Ruijin Hospital, Shanghai Jiao Tong University School of Medicine, Shanghai 201821, China*

**DATA AVAILABILITY**

The raw scRNA sequencing data from tumor samples could be accessed at GEO (accession number: GSE152048), MPE samples were deposited at the National Genomics Data Center/China National Center for Bioinformation (CNBC-NGDC; accession number: CNP0003329). The TARGET-osteosarcoma transcriptomic sequencing data and the corresponding clinical information could be accessed at https://ocg.cancer.gov/programs/target/data-matrix. All the R scripts supporting this study are available from the corresponding author upon reasonable request.

1. [↑](#footnote-ref-1)
