# Supplementary material for: Characterization of the tumour microenvironment phenotypes in malignant tissues and pleural effusion from advanced osteoblastic osteosarcoma patients
Source: Clin Transl Med. 2022 Oct 28;12(11):e1072. doi: 10.1002/ctm2.1072 (PMC9615475; doi:10.1002/ctm2.1072)
Supplement: Supplementary file 3 — Supplementary material [file CTM2-12-e1072-s004.docx]

**Table S1.** Clinical characteristics of the osteosarcoma patients recruited in scRNA-seq analysis

| Sample | Gender | Age | Type | Location | Preoperative chemotherapy | Size (cm) | Necrosis rate | Ki67+ |
| --- | --- | --- | --- | --- | --- | --- | --- | --- |
| BC2 | M | 11 | Primary | Femur | 4 times (MTX, AP, IFO, MTX) | 5.5*5*3 | < 90% | 50% |
| BC3 | F | 11 | Primary | Tibia | 6 times (MTX, AP, MTX, AP, MTX, MTX) | 8*6*6 | < 90% | 70% |
| BC5 | F | 19 | Primary | Femur | 3 times (MTX, IFO, AP) | 8*7.5*6 | ≥ 90% | 80% |
| BC6 | F | 23 | Primary | Ulna | 3 times (MTX, IFO, AP) | 7*7*4 | ≥ 90% | 15% |
| BC10 | F | 19 | Metastasis (Lung) | Femur | 2 times (GT, GT) | 3.5*3*2 | < 90% | 8% |
| BC11 | M | 12 | Recurrent | Femur | 3 times (GT, GT, GT) | 20*11*10 | < 90% | 30% |
| BC16 | M | 11 | Primary | Tibia | 4 times (IFO, AP, MTX, MTX) | 6*4*2.5 | < 90% | 40% |
| BC21 | F | 38 | Primary | Femur | 4 times (MTX, AP, IFO, AP) | 5*4*1 | < 90% | 10% |
| MPE28 | F | 26 | Metastasis (Lung) | Tibia | first line 5 times (MAPI sequential treatment) | 7.5*6*2.5 | < 90% | 30% |
| MPE29 | F | 56 | Metastasis (Lung) | [sternum](http://dict.youdao.com/w/sternum/#keyfrom=E2Ctranslation) | first line 5 times (MAPI sequential treatment) + second line 1 times GT | 8.3*4.7*2.5 | < 90% | 25% |
| MPE32 | M | 32 | Metastasis (Lung) | Femur | first line 4 times (MTX, AP, IFO, AP) + second line 6 times GT + third line 7 times CPT-11+ aptinib | 11.8*8.4*5.5 | ≥ 90% | 30% |
| MPE33 | M | 13 | Metastasis (Lung) | Femur | first line 6 times (MAPI sequential treatment) + second line 6 times GT + aptinib for 17m | 15*8*6.5 | ≥ 90% | 55% |
| MPE34 | F | 31 | Metastasis (Lung) | Femur | first line 6 times (MAPI sequential treatment) + second line 8 times GT+ aptinib  third line 5times CPT-11+ anlotinib | 10.6*7.8*7.5 | ≥ 90% | 45% |
| MPE35 | F | 26 | Metastasis (Lung) | Tibia | 6 times (MAPI sequential treatment) | 11.6*6.7*4.6 | < 90% | 40% |
| MPE36 | M | 32 | Metastasis (Lung) | [humerus](http://dict.youdao.com/w/humerus%20(pl.%20humeri)/#keyfrom=E2Ctranslation) | first line 2 times (IFO, AP) | 8.9*6.3*7.2 | < 90% | 35% |

**Abbreviations:** MAPI including MTX, Methotrexate; AP, Doxorubicin + Cisplatin; IE, IFO + VP-16; IFO, Ifosfamide; GT, Gemcitabine + Docetaxel; VP-16, Etoposide.
