# Supplementary material for: Characterization of the tumour microenvironment phenotypes in malignant tissues and pleural effusion from advanced osteoblastic osteosarcoma patients
Source: Clin Transl Med. 2022 Oct 28;12(11):e1072. doi: 10.1002/ctm2.1072 (PMC9615475; doi:10.1002/ctm2.1072)
Supplement: Supplementary file 4 — Supplementary material [file CTM2-12-e1072-s002.docx]

**Table S2.** Summary of the genome-wide association studies of osteosarcoma susceptibility and prognosis

| **rsID** | **Gene** | **Outcome** | **Publication** |
| --- | --- | --- | --- |
| rs7023329 | MTAP | Risk | Br J Cancer. 2013 Apr 2;108(6):1378-86. |
| rs1906953 | GRM4 | Risk | Nat Genet. 2013 Jul;45(7):799-803. |
| rs7591996 | None | Risk | Nat Genet. 2013 Jul;45(7):799-803. |
| rs10208273 | None | Risk | Nat Genet. 2013 Jul;45(7):799-803. |
| rs9420907 | OBFC1 | Risk | Carcinogenesis. 2016 Jun; 37(6): 576–582. |
| rs7034162 | NFIB | Metastasis | Cancer Discov. 2015 Sep;5(9):920-31. |
| rs55933544 | GLDC | Overall survival | Int J Cancer. 2018 Apr 15;142(8):1594-1601. |
| rs55933544 | IL33 | Overall survival | Int J Cancer. 2018 Apr 15;142(8):1594-1601. |
